# Mitochondrial Transcription of Entomopathogenic Fungi Reveals Evolutionary Aspects of Mitogenomes

**DOI:** 10.3389/fmicb.2022.821638

**Published:** 2022-03-21

**Authors:** Stylianos P. Varassas, Vassili N. Kouvelis

**Affiliations:** Department of Genetics and Biotechnology, Faculty of Biology, National and Kapodistrian University of Athens, Athens, Greece

**Keywords:** mitochondrial (mt) transcription, entomopathogenic fungi, mt RNA polymerase, RPO41, mt transcription factor, MTF1, mt promoters

## Abstract

Entomopathogenic fungi and more specifically genera *Beauveria* and *Metarhizium* have been exploited for the biological control of pests. Genome analyses are important to understand better their mode of action and thus, improve their efficacy against their hosts. Until now, the sequences of their mitochondrial genomes were studied, but not at the level of transcription. Except of yeasts and *Neurospora crassa*, whose mt gene transcription is well described, in all other Ascomycota, i.e., Pezizomycotina, related information is extremely scarce. In this work, mt transcription and key enzymes of this function were studied. RT-PCR experiments and Northern hybridizations reveal the transcriptional map of the mt genomes of *B. bassiana* and *M. brunneum* species. The mt genes are transcribed in six main transcripts and undergo post-transcriptional modifications to create single gene transcripts. Promoters were determined in both mt genomes with a comparative *in silico* analysis, including all known information from other fungal mt genomes. The promoter consensus sequence is 5′-ATAGTTATTAT-3′ which is in accordance with the definition of the polycistronic transcripts determined with the experiments described above. Moreover, 5′-RACE experiments in the case of premature polycistronic transcript *nad*1-*nad*4-*atp*8-*atp*6 revealed the 5′ end of the RNA transcript immediately after the *in silico* determined promoter, as also found in other fungal species. Since several conserved elements were retrieved from these analyses compared to the already known data from yeasts and *N. crassa*, the phylogenetic analyses of mt RNA polymerase (Rpo41) and its transcriptional factor (Mtf1) were performed in order to define their evolution. As expected, it was found that fungal Rpo41 originate from the respective polymerase of T7/T3 phages, while the ancestor of Mtf1 is of alpha-proteobacterial origin. Therefore, this study presents insights about the fidelity of the mt single-subunit phage-like RNA polymerase during transcription, since the correct identification of mt promoters from Rpo41 requires an ortholog to bacterial sigma factor, i.e., Mtf1. Thus, a previously proposed hypothesis of a phage infected alpha-proteobacterium as the endosymbiotic progenitor of mitochondrion is confirmed in this study and further upgraded by the co-evolution of the bacterial (Mtf1) and viral (Rpo41) originated components in one functional unit.

## Introduction

Mitochondria are the semiautonomous powerhouses of the cell that carry their own small genome that does not encode for all the products needed by mitochondria (Burger et al., 2003). Especially in fungi, mitogenomes show such a great diversity regarding genome size, intergenic regions and intron content (Mukhopadhyay and Hausner, 2021; Yildiz and Ozkilinc, 2021) that can be exploited for species and strain typing (Kortsinoglou et al., 2019, 2020) and other phylogenetic and evolutionary studies (Hausner, 2003; Aguileta et al., 2014; Fonseca et al., 2020; Glare et al., 2020; Megarioti and Kouvelis, 2020). However, almost within all fungal mt genomes, genes encoding rRNA (*rns* and *rnl*), tRNAs (*trn*) and proteins involved in mitochondrial ribosome assembly (*rps*3) and oxidative phosphorylation [i.e., subunits of ATP synthase (atp6, 8 and 9), apocytochrome b (*cob*) cytochrome c (*cox*1-3) and NADH dehydrogenase (*nad*1-6 and *nad*4L)] are commonly found (Paquin et al., 1997; Kouvelis et al., 2004). In only two cases this gene content is extensively different. Specifically, in several fungal species *rps*3 is missing or located in the nuclear genome (Korovesi et al., 2018) and *nad* genes cannot be found in the mitogenomes of the family Saccharomycetaceae of Saccharomycotina (Christinaki et al., 2021).

Therefore, it is expected that all functions of the mitochondria, like the transcription of their mt genes, to remain conserved. Until now, knowledge for this function in fungal mt genomes relies mostly on studies performed in yeasts and fungal model organisms like *Neurospora crassa* and *Schizosaccharomyces pombe* (Burger et al., 1985; Kleidon et al., 2003; Schäfer, 2005; Schäfer et al., 2005). In detail, mt transcription requires promoters which are participating in the simultaneous transcription of several genes in one transcript (polycistronic transcripts), as a result of the endosymbiotic origin of the mitochondria from a-proteobacteria, in which their genome is organized in operons under the regulation of one promoter (Martin et al., 2015). Based on yeasts studies, mt promoters contain an “A-T enriched” consensus sequence of 9 bp, which is recognized by the mitochondrial transcription machinery (Christianson and Rabinowitz, 1983; Schäfer et al., 2005; Deshpande and Patel, 2014). More specifically, in yeasts an mt promoter is characterized by the 5′-(−8)ATATAAGTA(+ 1)-3′ sequence, where + 1 represents the transcription start site (Biswas et al., 1985; Schinkel et al., 1986; Biswas, 1999; Kim et al., 2012). In *Neurospora crassa*, the mitochondrial promoter has a modified AT-rich consensus sequence, i.e., 5′- TTAG(A/T)RR(G/T)(G/C)N(A/T)-3′ (Kubelik et al., 1990; Kleidon et al., 2003). Mt promoters are dispersed throughout the mt genome. For example, in *Saccharomyces cerevisiae*, the mitochondrial transcription initiates in more than 10 different areas, each having this consensus as a promoter sequence (Costanzo and Fox, 1990; Pel and Grivell, 1993; Grivell, 1995; Gagliardi et al., 2004). After mt transcription produced the initial polycistronic transcripts, they are further processed and converted to monocistronic (mRNA, tRNA, rRNA) by being digested at the 5′ and 3′ untranslated regions (UTRs). This transcript maturation has been proven experimentally not only in *S. cerevisiae*, but additionally, in *Neurospora crassa* and *Schizosaccharomyces pombe* (Kennell and Lambowitz, 1989; Kubelik et al., 1990; Dieckmann and Staples, 1994; Schäfer, 2005). Whenever an intron was found within the mt gene, introns were self-spliced (Cech, 1990; Michel and Ferat, 1995), leading, thus, the whole transcription procedure to the final maturation of the mt RNAs, i.e., “single intronless gene transcript.” The mechanism of transcription termination in fungal mt genomes is not known (Lipinski et al., 2010), and only in vertebrates (Clayton, 1991), there are specific transcription termination sequences to enable the release of the transcript from the mtDNA. Alternatively, the formation of stem-loop structure in the native mitochondrial RNA and/or tRNAs might play the signal of transcription’s termination (Breitenberger et al., 1985; Christianson and Clayton, 1988; Clayton, 1991; Schäfer et al., 2005). The mature transcripts bear no cap nor polyadenylation (Rorbach et al., 2014).

Regarding the most important proteins implicated in fungal mitochondrial transcription, two proteins, i.e., mtRNA polymerase (mtRNAP or Rpo41) and Mtf1 play the most significant roles (Lipinski et al., 2010; De Wijngaert et al., 2021). Both proteins are encoded by nuclear genes, i.e., *rpo*41 and *mtf*1, as the former is the catalytic component of the transcription and the latter is the sole needed transcription factor for the orderly function of the polymerase, in contrast to the nuclear polymerases for which many different transcription factors are needed (Yang et al., 2015). It is known that RNA polymerase (mtRNAP) is homologous with the respective polymerase of bacteriophage T3/T7 (Masters et al., 1987; McAllister and Raskin, 1993; Filée and Forterre, 2005). Unlike the RNAP of the T7 phage, which is a single subunit RNA polymerase (ssuRNAP) that alone catalyzes all stages of transcription, mtRNAP is dependent from transcription factors for the initiation of this process (Bonawitz et al., 2006; Jiang et al., 2011). Similarly, archaeal, and fungal nuclear RNA polymerases comprise many modules (multi-subunit RNAP; msuRNAP) and depend on a large number of transcription factors (Borukhov and Severinov, 2002). Rpo41 in yeasts consists of a single enzyme subunit, divided into several domains, including the C-terminal domain (CTD), the N-terminal domain (NTD), and the N-terminal extension (NTE) (Rodeheffer et al., 2001; Deshpande and Patel, 2012). The Mtf1 is a compact protein of 43 kDa, that has an N-terminal region, C-terminal region and a flexible C-terminal tail (Schubot et al., 2001; Deshpande and Patel, 2012). The C-terminal tail is very important as it interacts with the promoter during the creation of the transcription initiation complex (Savkina et al., 2010). The N-terminal domain contains the S-adenosyl-methionine (SAM)—binding site, which is usually found in methyltransferases, although the SAM-binding site is not essential for transcription (Cotney and Shadel, 2006). Nevertheless, the mt transcription pre-initiation complex of Rpo41 and Mtf1 plays an active role in the melting of the DNA and the local denaturation of the promoter region (Sologub et al., 2009; Deshpande and Patel, 2014; Ramachandran et al., 2016). The denatured nucleotides from mtRNA polymerase’s activity can be found at positions −4 to + 2 in the promoter of the mitochondrial gene (Paratkar and Patel, 2010; Velazquez et al., 2015; Basu et al., 2020).

While the majority of research on mt genome diversity and its functions has been focused on yeasts (e.g., Freel et al., 2015; Kolondra et al., 2015), it is important to expand further the studies to other subphyla of Ascomycota, specifically in the Pezizomycotina (Pantou et al., 2008) in order to fully clarify the similarities of the mt gene expression mechanisms. Entomopathogenic fungi like the Hypocrealean species *Metarhizium brunneum* and *Beauveria bassiana* may act as model organisms for studying the transcription mechanism of mt genes for several reasons. Firstly, their mt genomes are already known and analyzed (Ghikas et al., 2010; Kortsinoglou et al., 2020) in addition to their whole genomes (Gao et al., 2011; Saud et al., 2021). Secondly, both species have been used as Biological Control Agents (BCAs), and thus, they have been proposed as safe alternatives for the protection of several different crops worldwide (Butt et al., 2001; Typas and Kouvelis, 2012). Gaining insights into the functional mechanisms of their mt genomes may provide a starting point for future genetic modifications of these genomes, with a final aim of improving the efficacy of their entomopathogenic activity against the pests which destroy the crops.

The scientific and economical interest for exploiting these two entomopathogenic fungi (Gao et al., 2011; Kouvelis et al., 2011; Xiao et al., 2012), the need for better knowledge of BCAs (Clarkson and Charnley, 1996; Strasser et al., 2000; Vey et al., 2001; Shahid et al., 2012; Butt et al., 2016; Lovett and Leger, 2016) and the lack of genetic data at the mitochondrial genome processes of the above entomopathogenic fungi (Kouvelis et al., 2004; Ghikas et al., 2006, 2010) were the motivation for studying the mechanisms and prime enzymes of fungal mitochondrial transcription (mtRNA processing). Therefore, in this study the promoters of the mt genomes for these two Hypocrealean species and their polycistronic transcripts will be determined. In addition, the phylogenetic evolution of the two nuclear encoded proteins that are crucial for the mt transcription, like the mtRNA polymerase (Rpo41) and its transcription factor Mtf1, will provide important knowledge through the comparative analysis with the already known data from yeasts. Thus, the evolution of a mitochondrial process like transcription will be examined from both the “structural” (promoters, transcripts, maturation of polycistronic transcripts) and the “functional” (key protein enzymes involved, like Rpo41 and Mtf1) aspects in order to create a hypothesis which explains the current form of mt transcription.

## Materials and Methods

### Strains Used, Growth Conditions and DNA/RNA Extraction

*Metarhizium brunneum* strain ARSEF 3297 (from USDA-ARS Collection of Entomopathogenic Fungal Culture, Ithaca, NY, United States) and *Beauveria bassiana* strain ATHUM 4946 (from ATHUM culture collection of fungi, Athens, Greece) were used for determining the primary polycistronic transcripts of their mitochondrial genomes. Fungi were grown on a rich complete Potato Dextrose Agar (PDA) liquid medium, with the addition of 2 gl^–1^ casein hydrolyzate, malt extract, yeast extract, and mycological peptone (Kouvelis et al., 2008a). For solid media, 1.5% agar was added. All strains used were derived from single conidia grown on PDA plates (11 days old). Shake flask cultures were grown at 25°C and after 4–5 days, mycelia were collected by vacuum filtration, lyophilized for 4 days and crushed in liquid nitrogen using a mortar and pestle. Lyophilized, ground mycelia were maintained at −80°C. Isolation of total genomic and mitochondrial DNA was performed as previously described (Kouvelis et al., 2008b). Total cellular RNA was isolated from 50 to 100 mg lyophilized, grounded mycelium using the TRIzol™ Reagent (Invitrogen, Waltham, MA), following the manufacturer’s instructions. Total RNA was eluted in 150 μl of RNase free water (DEPC-treated), treated with DNAse I and stored at –80°C.

### Mitochondrial Genome Annotation and Analysis

The complete nucleotide sequence of the mitochondrial DNA from the entomopathogenic fungus *Metarhizium brunneum* ARSEF3297 appears in the GenBank Nucleotide database under accession number NW_014574670.1 (Contig: AZNG01000047) (Hu et al., 2014). In this work, the mitochondrial genome of strain ARSEF3297 was retrieved and annotated (Supplementary Figure 1 and Supplementary Table 1) as described previously (Ghikas et al., 2006). Specifically, the protein coding and the ribosomal (rRNA) genes were identified using BLASTx and BLASTn (Altschul et al., 1990), respectively, after comparisons with known related sequences and mostly with the other known mt genome of *M. brunneun* ARSEF 4556 (Supplementary Table 2; Kortsinoglou et al., 2020). The *trn* genes were detected using the online software tRNAScan-SE (Chan and Lowe, 2019) and intron characterization was performed using RNAweasel and MFannot (Lang et al., 2007).

### *In silico* Prediction of Promoters

The complete mt genomes of *M. brunneum* ARSEF 3297 (the leading strain of this work), *M. brunneum* ARSEF 4556 (Kortsinoglou et al., 2020), *M. acridum* CQMa102 (Gao et al., 2011), and of *B. bassiana* Bb147 (Ghikas et al., 2010) were aligned using MegAlign program of Lasergene-DNASTAR suite by employing ClustalW (Larkin et al., 2007) with default parameters. Emphasis was given in the 3′ end of intergenic regions at the 5′ end of all mt genes in order to retrieve the conserved regions. In addition, the presence of the suggested 9 bp mt promoter of *Saccharomyces cerevisiae*, which is known as non-anucleotide (Osinga et al., 1982), along with the proposed mt promoter sequences of other fungal species, i.e., *Neurospora crassa* (Kennell and Lambowitz, 1989; Kubelik et al., 1990; Kleidon et al., 2003), *Schizosaccharomyces pombe* (Schäfer, 2005; Schäfer et al., 2005), *Candida albicans* (Kolondra et al., 2015), and *Starmellera bacillaris* (Pramateftaki et al., 2006) were searched manually as they were helpful for defining the putative promoter sequences of the mt genomes of these entomopathogenic fungi belonging to the order Hypocreales.

### *In silico* Search for Termination Sequences of Transcription Units

To determine the presence of termination sequences for the mt transcription units, the nucleotide sequences downstream each mt transcription unit of both species were retrieved and aligned with Clustal Omega program (Sievers and Higgins, 2021). In comparison with the known yeast mt termination sequences (Osinga et al., 1984; Butow et al., 1989), no similar sequences were found. Thus, a comparative search among the different intergenic regions was performed to find the conserved termination sequences in the aligned matrices.

### Protein Molecular Modeling

The sequence of Rpo41 of *M. brunneum* was retrieved from the whole genome (Nucleotide Acc No. NW_014574670.1) and primers were designed at the 5′ and 3′ end of the gene (Table 1). The amplicon was cloned and sequenced in both directions in order to verify the sequence before analyzing its secondary structure. Prediction of the Rpo41 secondary structure was performed using the PSIPRED 4.0 Workbench (UCL-CS Bioinformatics, London, United Kingdom) (Buchan and Jones, 2019). For homology modeling of the Rpo41 protein, the Hidden Markov Model-based tool HHPred (Zimmermann et al., 2018) and MODELLER 9.25 (Webb and Sali, 2016) were used, based on the highly similar crystal structure of yeast mitochondrial RNA polymerase from *S. cerevisiae* (Protein Data Bank, PDB 6YMW) with 98.94% probability and 1.4 × 10^–21^ *E*-value, as described previously (Deshpande and Patel, 2012; De Wijngaert et al., 2021). Protein structures were visualized and compared using PyMOL 2.4.^1^

**TABLE 1:** Oligonucleotide primers used for PCR and RT-PCR assays in this work.

Primer name	Primer sequence (5′3′)	Length (bp)	T m (°C)	GC (%)	PCR product (bp)
nad1UF	5′-TATGCAAAGAAGAATAGGTCCAAAT-3′	25	52.6	32	560
nad1UR	5′-TAACCACCTAAGAATAAAATAGTAG-′	25	44.2	28	
nad1b	5′-GCATGTTCTGTCATAAASCCACTAAC-3′	26	53.1	42.3	
cox1UF	5′-CAATTATACAATGTTATAGTTACTGCTCAT-3′	30	51.0	26.7	600
cox1UR	5′-CCAAAACCAGGTATAATTACAATATA-3′	26	48.7	26.9	
cox2UF	5′-GCTTTCCCTTCATTTAATTTATTGTATTTA-3′	30	55	23.3	350
cox2UR	5′-AAGCAGATGCTTGATTTAATCTACCAG-3′	27	55.7	37	
cox3UF	5′-CATATAGTATCACCATCACCTTGACC-3′	26	52.8	42.3	460
cox3UR	5′-ACATGTAATCCATGGAAACCTG-3′	22	50.6	40.9	
cobUF	5′-GTTATTACTAATTTATTAAGTGCGTTCC-3′	29	50.2	27.6	400
cobUR	5′-GCATAGAAAGGTAGTAAGTATCATTC-3′	26	47.5	34.6	
rnsUF	5′-GCCAGCAGTCGCGGTAATAC-3′	20	54.8	60	600
rnsUR	5′-TATAAAGGCCATGATGTCTTGTCTT-3′	25	53.2	36	
atp6UF	5′-CATGGATTAGAATTCTTCTCATTA-3′	24	47.2	29.2	200
atp6UR	5′-ATATTAGCAGCTAAACTAAAACCTA-3′	25	46.3	28	
atp8UF	5′-ATGCCACAATTAGTACCATTTTA-3′	23	48.2	30.4	800
nad2PF	5′-TATTATTAATATCTAGTAGTGATTTAGTATC-3′	31	44.1	19.4	400
nad2PR	5′-ACAAAAGTTGTAACAATTGTAGGTAT-3′	26	48	26.9	
nad4PF	5′-GATGGTATATCTATATATTCTGTATTATTGAC-3′	32	48.9	25	737
nad4PR	5′-CCTTCTATACCTTGTATTGTATTACTAAA-3′	29	48.8	27.6	
nad4LF	5′-GAGGAAGTATAGCAATAGAGTATAAATAA-3′	29	48.3	27.6	400
nad5PoR	5′-CATCATAAAAGTAAATAAAACTAAATA-3′	27	44.5	14.8	
nad5PF	5′-TATTTAAGTTTATTTACTTTTATGATG-3′	27	44.5	44.5	500
nad5PR	5′-ACTAAATACTGTAGTTATAGCACCTAATC-3′	29	48.1	31	
nad6PF	5′-GTATATATAGGAGCTGTATCAATCTTA-3′	27	45.6	29.6	337
nad6PR	5′-CACCTACCATAGCTAATAATAGAAT-3′	25	45.9	32	
rps3F	5′-AAAAATATTCCAAGCCATCAT-3′	21	47.7	28	400
rps3R	5′-TTAGTGCCTTCAAAAACATTAT-′	22	46.1	27	
trnAF	5′-AGGTTCGATTCCTAGTTTCTCC-3′	22	50.4	44	400
trnQR	5′-GTGATTCGAACACCCACTATTG-3′	22	51.5	45	
trnTF	5′-GGCGCGATACCTCCATT-3′	17	50	58	1,200
trnM2R	5′-TGACTTGAACACTTATTATTACTG-3′	24	43	34	
atp8UR	5′-TTATATATTATTAATAAACATACGTGATAAG-3′	31	47.1	26	200
atp9F	5′-ATGTTACAATCTTCAAAAATAATAGG-3′	26	48	23	200
atp9R	5′-TTAAGCAACATTTAATAATAATAATG-3′	26	45.5	25	
rnloF	5′-CTAAGTTGGTTAAGGATAAGTG-3′	22	43	36	800
rnloR	5′-CATTTCTTTTCCTGAAGTTAC-3′	21	43.1	33	
nad4LFN	5′-GAATTTTAGGATTCGTATTTAATAG-3′	25	46	26	250
nad4LRN	5′-TTATTTATATTCTATTGAGATACTTCC-3′	27	45	28	
rnlF	5′-CAAAAGATATCAAAAGAGATTC-3′	22	42.6	27.8	600
rnlR	5′- CACTTATCCTTAACCAACTTAG-3′	22	43	36.4	
nad3F	5′- ATTTGAATGTGGTTTTCAT-3′	19	41.3	26.3	250
nad3R	5′- AATGCASTTTTACCTAATTCA-3′	21	44.3	28.6	
Rpo41F	5′-GCAAGATGCTCAGTCGACAAACAAGGAG-3′	28	64.5	48.3	4,300
Rpo41R	5′-GCTCAATAAAATACACCAAATGCCAACTC-3′	29	60.9	36.7	

### Polymerase Chain Reaction Amplification and Cloning

Mitochondrial gene fragments for seven mt genes (*rnl*, *rps*3, *nad*3, *cob*, *cox*1, *nad*4, and *cox*3) were amplified by PCR to produce probes for Northern hybridization analyses. Newly designed primers, as well as primers proposed by Kouvelis et al. (2004) and Ghikas et al. (2006) were used (Table 1) and 1 μl total DNA (1 μl) from each strain was used as template. Polymerase chain reaction (PCR) amplifications were performed with Taq DNA polymerase (Invitrogen, Waltham, MA), in a FastGene^®^ Ultra Cycler Gradient (Nippon-Genetics Europe GmbH, Dueren, Germany), following previously described protocols (Kouvelis et al., 2004). Amplicons were separated in 1.5% agarose–TAE gels and amplicons’ purification was performed afterward, using Monarch^®^ PCR and DNA Cleanup Kit (New England Biolabs, Hitchin, United Kingdom), NucleoSpin™ Gel and PCR Clean-up Kit (Macherey-Nagel, Dueren, Germany). The vector pGEM^®^-T was used along with the pGEM^®^-T Vector System (Promega, Madison, WI) for the cloning. All recombinant plasmids were screened to verify that the insert’s size coincided with the amplicon’s size and three randomly chosen recombinant plasmids were sequenced. All fragments were sequenced in both directions and since sequences were identical (100% id) with the those found in the complete genomes of both *B. bassiana* and *M. brunneum*, they were not submitted to any of the publicly available databanks like GenBank or ENA of EMBL-EBI.

### Reverse Transcription-Polymerase Chain Reaction

The isolated total RNA (1 μg) was reverse transcribed to generate single-stranded complementary DNA (cDNA) using Moloney Murine Leukemia Virus Reverse Transcriptase (M-MLV RT) with reduced RNase H activity (Conc. 200 U/μl, SuperScript™ II Reverse Transcriptase, Invitrogen, Waltham, MA), according to the manufacturer’s instructions. For the analysis of multiple target mitochondrial RNAs, each first-strand cDNA was synthesized by priming with a gene-specific primer (Table 1). The cDNAs were stored at −80°C. After reverse transcription (RT), the cDNAs from different target RNAs were used as a template for amplification with specific primers by PCR (KAPA HiFi HotStart ReadyMix PCR Kit, Merck KGaA, Darmstadt, Germany). A negative control (DNAse-treated RNA, no reverse transcription) did not produce an amplification product in the PCR, confirming the cDNA (and not the mitochondrial DNA) origin of the PCR product. DNA fragments generated with KAPA HiFi HotStart DNA Polymerase (15 s extension per cycle for targets ≤ 1 kb, and 45 s/kb for longer fragments) have been used directly for blunt-end cloning. PCR products were cloned in vector pBluescript II SK (Stratagene, Agilent Technologies, Santa Clara, CA), subcloned as smaller fragments to pTZ57R/T cloning vector (only for large pcr fragments > 2 kb) and sequenced in both directions using the M13 Forward and Reverse universal primers. Amplicon sequences were analyzed using the «Sequence Scanner v.2» software (Thermo Fisher Scientific Inc., Waltham, MA). Additionally, the RT-PCR amplicons were separated on a 1,2% (w/v) agarose gel containing ethidium bromide and visualized under UV light (UVP Gel Imaging System, Thermo Fisher Scientific Inc., Waltham, MA).

### Northern Hybridizations

For Northern hybridization analysis, total cellular RNA (30 μg/lane) was electrophoresed in 1.5% formaldehyde-agarose gels and transferred to positively charged nylon filters. Specifically, RNA were resuspended in 50% deionized formamide, 10 mM morpholinepropanesulfonic acid (MOPS) pH 7.0, 1.4 mM sodium acetate, 0.5 mM EDTA, and 2.2 M formaldehyde. The RNA was denatured at 65°C for 15 min and fractionated on 1.5% agarose gel prepared in 1x formaldehyde gel running buffer (FGRB) with 2.2 mol/l formaldehyde, based to the protocol provided by DIG-High Prime DNA Labeling and Detection Starter Kit I (Roche Diagnostics, Sigma-Aldrich Inc., Darmstadt, Germany). The integrity of the electrophoresed total RNA was monitored by ethidium bromide staining of formaldehyde-agarose gel. The separated RNA fragments were then blotted onto a nylon filter (Porablot™ NY plus, Macherey-Nagel, Dueren, Germany) with 20x SSC (3 mol/l sodium chloride and 0.3 mol/l sodium citrate, pH 7.0) by applying a low pressure vacuum (VacuGene XL Vacuum Blotting System, Amersham Biosciences Corp., Buckinghamshire, United Kingdom). The filter was allowed to dry at room temperature for at least 30 min and the blotted RNA was immobilized on the filter by UV cross-linking. DIG-labeled probes were used for hybridization to blotting membranes as defined in standard methods (DIG-High Prime DNA Labeling and Detection Starter Kit I, Roche Diagnostics, Sigma-Aldrich Inc., Darmstadt, Germany). Mitochondrial DIG-labeled DNA probes (rnl, rps3, nad3, cob, cox1, nad4, cox3) were generated with DIG-High Prime solution according to the random primed labeling technique. Each northern blot was pre-hybridized with an appropriate volume (10 ml/100 cm^2^ filter) of DIG Easy Hyb buffer (5x SSC, 50% formamide deionized, 0,1% sodium-lauroylsarcosine, 0.02% SDS, and 2% Blocking Reagent) to hybridization temperature for 30 min with gentle agitation in an appropriate container (Hybaid H-9360 Hybridization Oven, Marshall Scientific LLC., Hampton, NH). Denatured DIG-labeled DNA probe were added to pre-heated DIG Easy Hyb buffer and hybridization was performed for 16hrs at 65°C (RNA). The RNA blots were washed with 2x SSC, 0.1% SDS for 25 min at room temperature under constant agitation. Subsequently, membranes were used directly for detection of mitochondrial polycistronic transcripts. The hybridized DNA probes were immunodetected with anti-digoxigenin-AP (Fab fragments) and were then visualized with the colorimetric substrates NBT/BCIP. Immuno−colorimetric detection was performed as described by DIG DNA Detection Kit. Results of Northern Blot Analysis were documented by photocopying the wet filter (BioSpectrum^®^ Imaging System, UVP Gel Imaging System, Thermo Fisher Scientific Inc., Waltham, MA).

### RACE Polymerase Chain Reaction

The 5′ end UTR of mt transcripts of a single polycistronic transcript has been determined using 5′-RACE (see section “Results”). Lyophilized, ground mycelium (100 mg) of the wild-type strains *B. bassiana* and *M. brunneum* was used for extraction of total RNA, as previously described. DNA was removed from the RNA sample by treatment with DNase I (Takara Bio Inc., Shiga, Japan). Reverse transcription and PCR reactions were carried out using a 5’/3’ RACE Kit (2nd Generation, Roche Applied Science, Merck KGaA, Darmstadt, Germany). The sequence of PCR primers used for definition of the 5′-ends of all mitochondrial transcripts is given in Table 2. Amplicons were purified and sequenced as described previously. The 5′ end UTR sequences of primary mitochondrial polycistronic transcripts have been determined *in silico* (see section “*In silico* Prediction of Promoters”), since the sequences were identical to the respective genomic fragments of the complete mt genomes of *M. brunneum* ARSEF 3297 and *B. bassiana* Bb 147 (GenBank Acc Nos: NW_014574670.1 and EU100742.1, respectively).

**TABLE 2:** 5′RACE primers.

Oligo name	Oligo sequence (5′–3′)
Oligo d(T)-anchor primer	5′-GACCACGCGTATCGATGTCGAC TTTTTTTTTTTTTTTTV-3′
PCR anchor primer	5′-GACCACGCGTATCGATGTCGAC-3′
NAD1.pr.ex.R	5′-CCATAATAACCTACAGCATTAGGCCCTAATCTTC-3′

### Phylogenetic Analyses and Methods of Examining Co-evolution

Phylogenetic analyses of mitochondrial Rpo41 and Mtf1 homologs were performed with the inclusion of sequences from four different taxonomic domains (Viruses, Archaea, Bacteria and Eukaryota; Table 3 and Supplementary Table 3). Phylogenetic analyses of Rpo41 and Mtf1 proteins were done separately for each protein at first, and then the matrices were concatenated to a single matrix. The respective proteins from other species were collected after a BLASTp search and all protein sequences were aligned with Clustal Omega (EMBL-EBI). The alignments were transferred to the program PAUP 4.0b10 (Swofford, 2002) for the Neighbor Joining (NJ) method, keeping all the parameters at default values. The topologies of the obtained trees were assessed by the bootstrap method (NJ-BP) and the number of replicates was set to 1,000. Each NJ tree was presented using FigTree v1.4.3.^2^ The maximum likelihood (ML) method was further employed to construct trees using RaxML (version 8.2.9) (Stamatakis, 2014). The best protein substitution model was found using ProtTest v3 (Darriba et al., 2011). For the matrices of RNA Polymerases and their transcription factors, the WAG + I + G + F model was found as most suitable, while the concatenated matrix had the LG + G model. Alpha values were set to 2.993, 2.694, and 2.959 for the RNA polymerases, Transcription Factors and concatenated matrices, respectively. All other parameters were estimated by RaxML. Results were assessed using 100 bootstrap replicates.

**TABLE 3:** Distribution of transcription factors and RNA polymerase subunits used in the phylogenetic analyses from species belonging to all domains of life and their taxonomic classification.

	Taxonomy					
	Domain	Kingdom	Phylum	Subphylum	Class	Number of strains
T3/T7 RNAP	Viruses	Heunggongvirae	Uroviricota	−	Caudoviricetes	2
aTBP/rpoB	Archaea	Crenarchaeota	Crenarchaeota	−	Thermoprotei	45
		Euryarchaeota	Hadesarchaeota	−	Hadesarchaea	3
		Proteoarchaeota	Verstraetearchaeota	−	−	3
			Odinarchaeota			1
			Thorarchaeota			1
			Lokiarchaeota;			3
σ-factor/rpb2	Bacteria	−	Proteobacteria	−	Alphaproteobacteria	17
					Betaproteobacteria	12
					Gammaproteobacteria	8
		Terrabacteria	Firmicutes	−	Bacilli	4
					Clostridia	6
			Cyanobacteria—Melainabacteria	Cyanobacteria	Cyanophyceae	13
			Deinococcus-Thermus	−	Deinococci	6
mtf1/rpo41	Eukaryota	Fungi	Ascomycota	Pezizomycotina	Dothideomycetes	10/0/0/0
					Eurotiomycetes	9/0/0/0
					Leotiomycetes	9/0/0/0
					Sordariomycetes	10/2/0/0
TBP(SL1)-RPA2				Saccharomycotina	Saccharomycetes	3/0/22/25
				Taphrinomycotina	Pneumocystidomycetes	4/0/3/0
					Schizosaccharomycetes	5/0/7/3
			Basidiomycota	Agaricomycotina	Agaricomycetes	16/0/0/0
TBP-RPB2				Pucciniomycotina	Microbotryomycetes	1/0/0/0
				Ustilaginomycotina	Ustilaginomycetes	1/0/0/0
			Early diverging fungal lineages	Mucoromycotina	Incertae cedis	3/0/0/0
TBP-RPC2		Metazoa	Chordata	−	Mammalia (Mammals)	2/41/21/25
				−	Reptiles	0/1/9/1
				−	Amphibia	0/29/0/1

In order to further examine the putatitve co-evolution of Rpo41 and Mtf1, three different methods were applied, STRING (Szklarczyk et al., 2021), Mirror Tree (Ochoa and Pazos, 2010), and Evolutionary Trace (Morgan et al., 2006). For both methods, STRING and ETviewer servers were used.^3^ ^,4^ In STRING method, the single proteins of *M. brunneum* and *S. cerevisiae* were used as input, under default parameters, while in the second method, Rpo41 (PDB: 6YMW) and Mtf1 (PDB: 1I4W) of *S. cerevisiae* were used. For the Mirror Tree, the method was employed through the server^5^ using as input the amino acid sequences from yeast. The comparison of the trees produced was impossible through the same server and thus, the single trees were obtained in Newick format and then compared with the phylo.io program (Robinson et al., 2016).^6^

## Results

### Mitochondrial Genome Annotation and Analysis

The complete mt genomes of both species, i.e., *B. bassiana* and *M. brunneum* used in this work are publicly available (Ghikas et al., 2010; Kortsinoglou et al., 2020). In this work, the strains used were ATHUM 4946 and ARSEF 3297, respectively. Since the whole genome sequence of the *M. brunneum* ARSEF 3297 is available, the mtDNA was retrieved and annotated by comparison to the known and annotated mt genome of strain ARSEF 4556 (Kortsinoglou et al., 2020) as described in “Materials and Methods”. Therefore, the mtDNA of this *M. brunneum* strain was found to be a circular molecule of 25,521 bp (Supplementary Figure 1 and Supplementary Table 1), with an overall G + C content of 28.8%. It contained all 14 protein encoding genes found in mtDNAs of the Pezizomycotina (Ghikas et al., 2006), the genes encoding for the large and small subunits of the ribosome (*rnl* and *rns*), and 25 genes encoding for tRNAs. A putative ribosomal protein (*rps*3) was also detected within the large ribosomal subunit using the *rps*3 homologs motif in fungal genomes (Bullerwell et al., 2000; Korovesi et al., 2018). In general, this mitogenome showed to be highly conserved to the genome of ARSEF 4556. Specifically, synteny was identical and the only variability could be observed in three intergenic regions. The ARSEF 3297 and ARSEF 4556 strains presented mt intergenic regions of sizes 4,069 and 4,215 bp, respectively. The largest intergenic region was that of *rns*-*trn*Y (496 bp) and the smallest was the 1bp overlap of genes *nad*4L-*nad*5. The identity of the intergenic sequences for both strains was 100% with the exception of the sequences that differentiated the size of the three intergenic regions, i.e., *nad*5-*cob*, *atp*6-*rns*, and *rns*-*trn*Y (with the addition of 198, 452, and 496 bp in ARSEF 3297).

### *In silico* Prediction of Promoters

**FIGURE 1:**
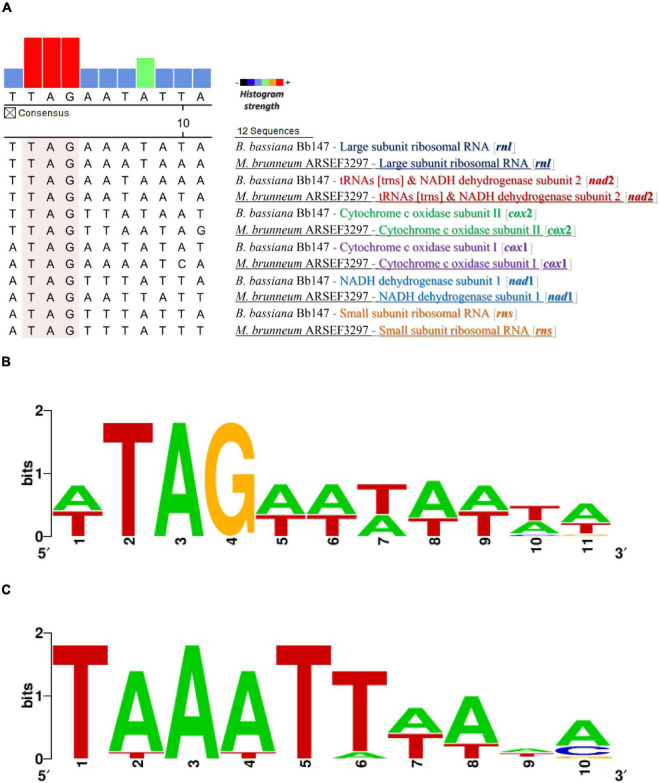
The promoter and transcription termination sequences as found from the *in silico* analyses. **(A)** Putative promoter sequence alignment for different mt genes from *M. brunneum* ARSEF3297 and *B. bassiana* Bb147. **(B)** The motif of the putative mt promoter produced by this alignment analysis. **(C)** The distinct putative mt transcription termination motifs that are located at a variable distance (1–218 nt) downstream from mRNA and rRNA coding regions in the genera *Metarhizium* and *Beauveria*.

The complete mt genomes of the species used in this work were searched for the existence of putative promoter sequences along with other known Hypocrealean mitogenomes based on known promoters from other fungal species as described previously in “Materials and Methods.” More emphasis was given to the intergenic regions of the complete mt genomes. It is worth mentioning that the intergenic regions were found to be highly enriched in A/T sequences. More specifically, the intergenic regions of the mt genomes of *B. bassiana* and *M. brunneum* presented A/T content of 73 and 76%, compared to the complete genomes’ A/T content of 72 and 71%, respectively. The search of possible repetitive elements did not present any conserved elements (data not shown), making, thus, the *in silico* identification of putative promoters extremely difficult. However, the consensus putative promoter sequence obtained by these comparative analyses was determined to be TTAGAATATTAT on the mitochondrial genomes of *Beauveria* and *Metarhizium* species (WTAGWWHWWHD: a modified consensus mt-promoter sequence within the order Hypocreales—data upon request) and this sequence was found scattered in six (6) intergenic regions (Figure 1).

### 3′ End Processing of Mitochondrial Transcripts

Most of the tRNA genes in *M. brunneum* ARSEF3297 and *B. bassiana* ATHUM4946 mitochondria are present as two major clusters upstream and downstream from the *rnl* gene, and are co-transcribed with mRNA sequences, according to the Northern Hybridizations. In at least two cases, initial transcripts (*cox*2-*trn*R1-*nad*4L-*nad*5-*cob*-*trn*C and *cox*1-*trn*R2) include tRNA sequences and are subsequently processed to generate the mature RNAs. The endpoints of these abundant mitochondrial transcripts generally coincide with those of tRNA sequences. We therefore conclude that tRNA sequences in some polycistronic transcripts act as primary signals for RNA processing in mitochondria of these entomopathogenic fungi (Punctuation model—single tRNAs may play this role). The situation is somewhat analogous to that observed in mammalian mitochondrial systems (Ojala et al., 1981; Rorbach and Minczuk, 2012) and in other filamentous fungi (*N. crassa*) (Breitenberger et al., 1985; Burger et al., 1985). In this study, the strongest evidence for tRNA sequences being primary signals in mitochondrial RNA processing comes from an analysis of transcription units 4 and 5 (*cox*1-*trn*R2 and *nad*1-*nad*4-*atp*8-*atp*6, respectively). Experimental results obtained by determining the 5′ end of the polycistronic transcript *nad*1-*nad*4-*atp*8-*atp*6 (5′ end RACE-PCR approach) indicated that *trn*R2 is co-transcribed with the gene *cox*1 (supported also by the Northern hybridization experiments). Furthermore, the 3′-termini of mRNAs and LSU rRNA are proposed to be distinct 5′-TAAATT-3′ motifs that are located at a variable distance (1–218 nt) downstream from mRNA and LSU-rRNA coding regions. Similarly, 3′-RNA processing motifs are also present in budding yeasts that have functionally analogous A + T rich dodecamer processing signals (Osinga et al., 1984; Butow et al., 1989; Hofmann et al., 1993).

### Defining the Polycistronic Transcripts

**FIGURE 2:**
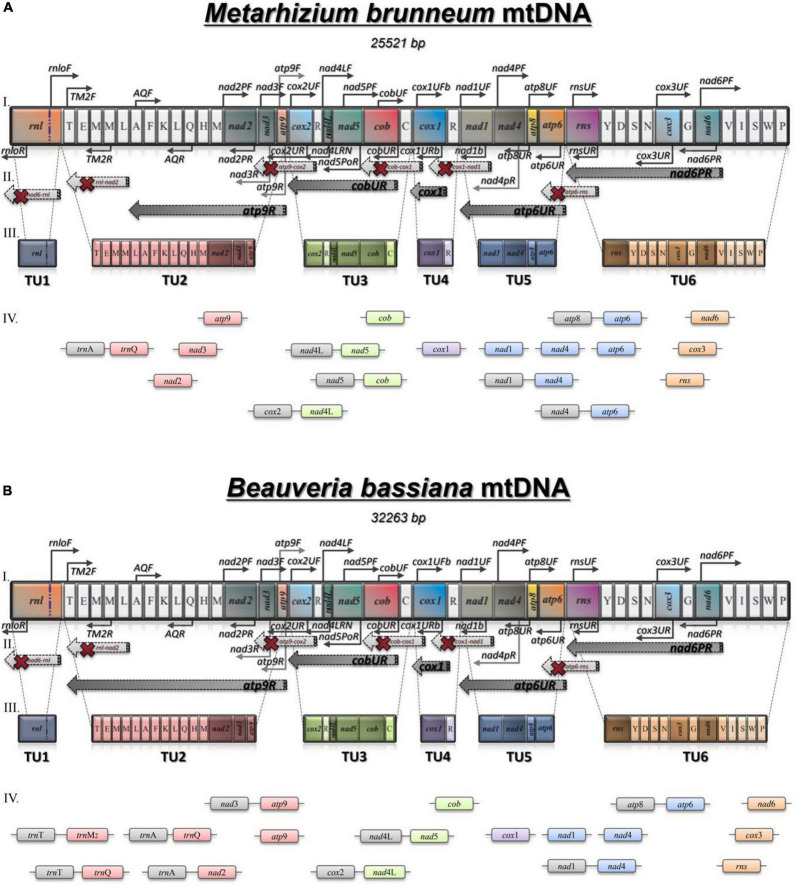
The mt transcripts of *M. brunneum* **(A)** and *B. bassiana* **(B)**: Schematic representation of the strategy followed to determine transcripts for both species: (i) The synteny of the mt genome. Black arrows above and below the genes of the mt genome show the primers used for the RT- PCR experiments. (ii) Dark gray arrows represent the successfully obtained RT-PCR amplicons, while light gray arrows with a red “X” represent RT-PCR efforts with no result. (iii) The block of genes included in primary transcripts as acquired by Northern hybridizations. (iv) The amplicons obtained with RT-PCR.

An RT-PCR strategy was developed to determine the number and content of premature polycistronic transcripts. The synteny of both mitogenomes of *M. brunneum* and *B. bassiana* are almost identical (with the differences located on the sequences of their intergenic regions; Figure 2). Therefore, the same sets of primers were designed and used for both fungal species, as described in “Materials and Methods.”

The amplified polycistronic transcripts were produced after amplification of cDNAs using pairs of primers for neighboring genes. This strategy, showing all combinations of primers (both successful and failed ones) in order to determine the polycistronic units is shown in Figure 2. After the completion of all RT-PCR experiments it became evident that there were 6 major pre-mature polycistronic transcripts as shown in Table 4.

**TABLE 4:** Pre-mature polycistronic transcripts and their sizes as produced by the RT-PCR experiments for both *B. bassiana* and *M.* anisopliae.

A/A	Gene composition of the polycistronic transcripts	Size (Kb)	
		Bb*	Mb*
I	***rnl*** (***rps*3**)	∼5	∼4.7
II	*trn*T-*trn*E-*trn*M1-*trn*M2-*trn*L1-*trn*A-*trn*F-*trn*K-*trn*L2-*trn*Q-*trn*H-*trn*M3-*nad*2-***nad*3**-*atp*9	∼4.5	∼3.5
III	*cox*2-*trn*R1-*nad*4L-*nad*5-***cob***- *trn*C	∼6	∼5
IV	***cox*1**- *trn*R2	∼4.5	∼2
V	*nad*1-***nad*4**-*atp*8-*atp*6	∼6	∼4
VI	*rns*-*trn*Y-*trn*D-*trn*S-*trn*N-***cox*3**-*trn*G-*nad*6-*trn*V(*trn*L)-*trn*I-*trn*S2-*trn*W-*trn*P	∼5 kb	∼5.5

The mt genes or parts of the genes of M. brunneum which have been used as probes in the Northern hybridization experiments in bold. *Mb, M. brunneum ARSEF3297, Bb, B. bassiana ATHUM4946.

**FIGURE 3:**
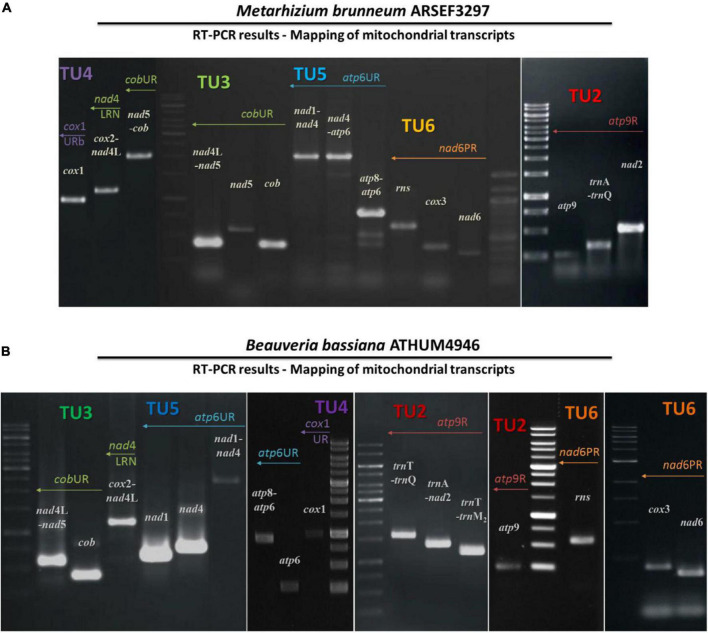
The amplicons of mitochondrial primary transcripts as obtained by RT-PCR followed by PCR in the entomopathogenic fungi *M. brunneum* ARSEF3297 **(A)** and *B. bassiana* ATHUM 4946 **(B)**. The actual amplicons obtained. Ladder 1kb (GeneRuler 1 kb DNA Ladder, Thermo Fisher Scientific Inc, Waltham, MA) was used as marker. TU, transcription unit.

Based on these RT-PCR results from both entomopathogenic fungi, it is worth mentioning that the cDNA generated with primer cox1URb produced a monocistronic transcript for *cox*1 gene, but it is possible that this gene can be co-transcribed with *trn*R2 as shown from the Northern hybridization experiments and the expected by size band (Supplementary Figure 2). The effort to amplify the *cob*-*cox*1 region did not produce any amplicon, indicating that the transcription of these two genes occurs in different polycistronic molecules. However, *cob* is co-transcribed with *nad*5, as shown in the case of *M. brunneum*, where a 2.1 Kb *cob*-*nad*5 amplicon is retrieved from the cDNA of cobUR (Table 5 and Figures 2, 3) and the amplification of both *nad*5 and *nad*4L-*nad*5 regions are successful for both species (Table 5 and Figures 2, 3). Moreover, neither *nad*3 nor *atp*9 gene could be identified from cDNA generated by primers nad4LRN and cox2UR, suggesting that this set of genes is transcribed separately from the *cox*2-*trn*R-*nad*4L-*nad*5-*cob* primary transcript (Figure 2). Additionally, by using either primers atp9R or nad2PR for first strand cDNA, followed by PCR with primers amplifying *nad*2, *nad*3, *atp*9, and the regions *trn*T-*trn*Q (which contains tRNA genes for amino acids TEM_1_M_2_L_1_AFKL_2_Q) and *nad*3-*atp*9, amplicons were obtained (Table 5 and Figure 3). However, the region *rnl*-*nad*2 was not amplified, suggesting the existence of two different transcripts for these two regions (Figure 2). This implies that the *trn* gene cluster as well as genes *nad*2, *nad*3, *atp*9 are co-transcribed as a pre-mature unit in both strains (Figure 2). Solely based on RT-PCR, the tRNAs lying upstream of the above transcription unit are co-transcribed with genes *nad*2, *nad*3, and *atp*9. No PCR products were obtained with the primer pairs nad6PF/rnloR and cox3UF/rnloR with cDNA generated with RT primer rnloR (Table 5 and Figure 3). Considering the above-mentioned RT-PCR results, it became clear that *cox*3 and *nad*6 are transcribed separately from the *rnl*-*rps*3 transcription unit, which was further verified when the cDNA template of nad6PR was used successfully for the amplification of *rns*, *nad*6, and *cox*3 genes (Table 5 and Figure 3). Thus, *rns* and *cox*3 are co-transcribed with the adjacent *nad*6 gene. In view of all these data, co-transcription of *rns*-*trn*Y-*trn*D-*trn*S-*trn*N-*cox*3-*trn*G-*nad*6 was found. No PCR amplicon of either *atp*8 or *atp*6 was obtained from cDNA generated by primer rnsUR (Table 5 and Figure 2), showing the existence of two different transcripts for *rns* and *atp*8*-atp*6. The co-transcription of *atp*8 with *atp*6 was confirmed with the partial PCR amplification of the genes included in this cDNA, and with the amplification of the whole targeted sequence, when the cDNA of atp6UR was used as template. Similarly, based on the same cDNA, the corresponding single gene amplicons of *nad*1 and *nad*4 genes, as well as, the *nad*1-*nad*4 intergenic sequence were produced (Table 5 and Figures 2, 3). Finally, no product for the sequence between *cox*1-*nad*1genes was obtained from cDNA generated by primer nad1b (Table 5 and Figure 2). Thus, *cox*1 is transcribed to a different molecule than the polycistronic transcript which contains *nad*1-*nad*4-*atp*8-*atp*6 (Figure 2). Therefore, the RT experiments, showed the composition of all polycistronic transcripts with the exception of the five tRNA genes, i.e., *trn*V, *trn*I, *trn*S, *trn*W, *trn*P. For that reason, their inclusion to a polycistronic transcript was found by Northern blot hybridization analysis, while all other RT based results were further verified with Northern hybridizations (Table 4).

**TABLE 5:** Amplicons produced with RT-PCR experiments, and their sizes after the PCR amplification in kbs.

First-Strand cDNA Synthesis (Reverse primer)	Reverse transcription PCR (Primer Pair)	Gene or region amplified after 2nd PCR/length (kb)	Bb amplicon size (kb)	Mb amplicon size (kb)
cox1URb	cox1UFb-cox1URb	*cox*1	1	1
	cobUF-cox1URb	*cob*-*cox*1	ND	ND
cobUR	cobUF-cobUR	*cob*	0.45	0.45
	nad4LF-nad5PoR	*nad*4L-*nad*5	0.4	0.4
	nad5PF-cobUR	*nad*5-*cob*	NT	2.1
	nad5PF-nad5PR	nad*5*	0.5	0.5
cox2UR	nad3F-cox2UR	*nad*3-*cox*2	ND	ND
	atp9F-cox2UR	*atp*9-*cox*2	ND	ND
nad4LRN	atp9F-nad4LRN	*atp*9-*nad*4L	ND	ND
	cox2UF-nad4LRN	*cox*2-*nad*4L	1.1	1.1
nad2PR	TM2F-TM2R	*trn*T-*trn*M2	1.2	NT
	TM2F-AQR	*trn*T-*trn*Q	1.7	NT
	AQF-AQR	*trn*A-*trn*Q	0.5	0.5
	AQF-nad2PR	*trn*A-*nad*2	1.5	NT
	rnloF-nad2PR	*rnl*-*nad*2	ND	ND
atp9R	atp9F-atp9R	*atp*9	0.2	0.2
	nad2PF-nad2PR	*nad*2	NT	0.4
	nad3A-nad3B	*nad*3	NT	0.3
	nad3A-atp9R	*nad*3-*atp*9	0.75	NT
rnloR	cox3UF-rnloR	*cox*3-*rnl*	ND	ND
	nad6PF-rnloR	*nad*6-*rnl*	ND	ND
nad6PR	rnsUF-rnsUR	*rns*	0.6	0.6
	cox3UF-cox3UR	*cox*3	0.45	0.45
	nad6PF-nad6pR	*nad*6	0.4	0.4
rnsUR	nad4PF-rnsUR	*nad*4-*rns*	ND	ND
	atp8UF-rnsUR	*atp*8-*rns*	ND	ND
atp6UR	atp8UF-atp6UR	*atp*8-*atp*6	0.9	0.85
	atp6UF-atp6UR	*atp*6	0.25	0.25
	nad1UF-nad4PR	*nad*1-*nad*4	2.4	2.2
	nad1UF-nad1UR	*nad*1	0.55	0.55
	nad4PF-nad4PR	*nad*4	0.7	0.7
	nad4PF-atp6UR	*nad*4-*atp*6	NT	2
atp8UR	cox1UF-nad1UR	*cox*1-*nad*1	ND	ND
nad1b	cox1UFb-nad1b	*cox*1-*nad*1	ND	ND

*Bb and Mb denote B. bassiana and M. brunneum, respectively. ND, not determined; NT, non-tested.*

DIG-labeled DNA probes from mitochondrial gene fragments of *rnl*, *rps*3, *nad*3, *cob*, *cox*1, *nad*4, and *cox*3 were used for verifying the pre-mature transcripts (Table 4). In all cases, several bands were hybridized with the probes in both species (Supplementary Figure 2). However, the largest hybridized band corresponded to the expected size of the primary polycistronic transcripts, and the smaller bands may denote maturation products (Supplementary Figure 2), according to the mechanisms proposed for *S. cerevisiae*, *Neurospora crassa*, and *Schizosaccharomyces pombe* (Kennell and Lambowitz, 1989; Kubelik et al., 1990; Dieckmann and Staples, 1994; Schäfer, 2005).

### Determining the 5′ End of the Polycistronic Transcript *nad*1-*nad*4-*atp*8-*atp*6

**FIGURE 4:**
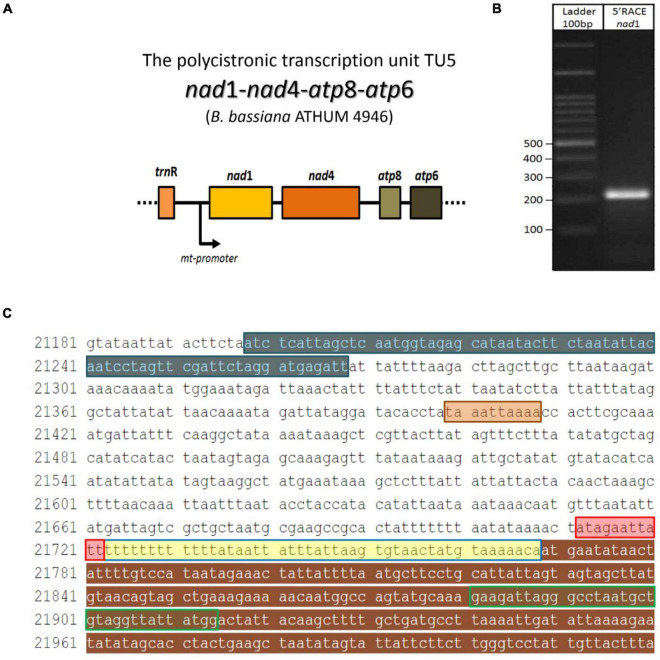
The 5′UTR region of the polycistronic transcript *nad*1-*nad*4-*atp*8-*atp*6 (unit V) in the mt genome of *B. bassiana* ATHUM 4946. **(A)** Schematic representation of the polycistronic transcript. The arrow indicated the position of the mt promoter. **(B)** The amplicon obtained after the RACE-PCR experiment. **(C)** The *trn*R2—*nad*1 sequence as retrieved from EU100742.1 whole mt genome of *B. bassiana*. Sequence regions of the amplicon: the *trn*R gene, the 5′ end partial *nad*1 gene, the promoter sequence and the 5′ UTR (as read by the 5′end RACE-PCR) are highlighted in blue, brown, red, and yellow, respectively. The primer nad1R which was used for the 2nd PCR of the RACE, is shown in green line box. The termination sequence of the previous transcript (Unit IV) is highlighted in orange.

Since the *in silico* putative promoters were further verified with the determination of the polycistronic transcripts, the approach of 5′ end RACE-PCR with subsequent sequencing of the obtained amplicon was selected in the case of transcript V (Table 2), as a sampling test to support further and combine both *in silico* and *in vitro* findings. This experiment showed that the 5′ end UTR of an mt transcript in *B. bassiana* is 46 bp upstream the ATG, which encodes the methionine of *nad*1 and is located immediately after the promoter of the polycistronic pre-mature transcriptional unit (Figure 4).

### The Mitochondrial Transcription RNA Polymerase Rpo41 and Its Transcription Factor MTF1

**FIGURE 5:**
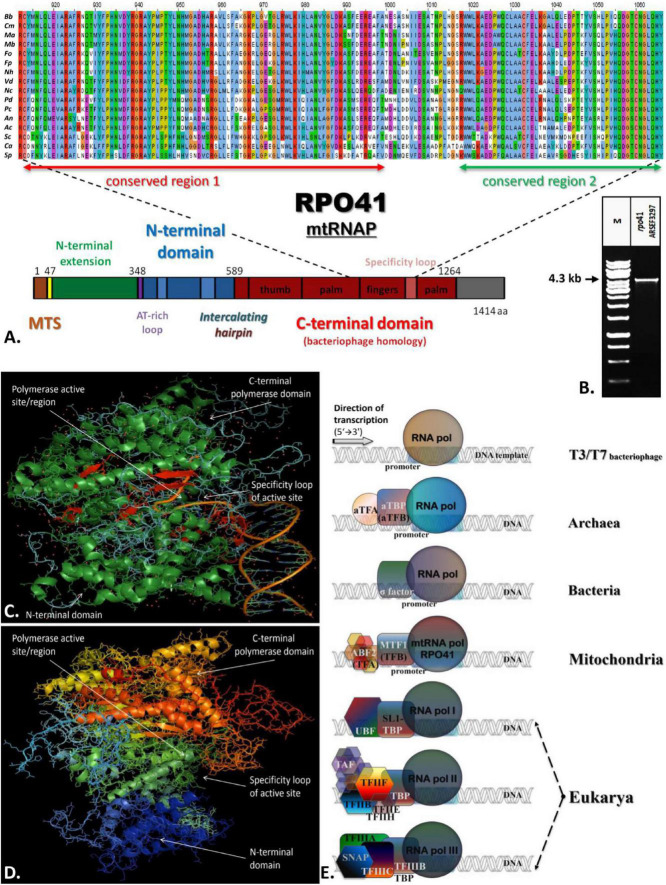
The structure of Rpo41 from *M. brunneum* ARSEF 3297. **(A)** Multiple sequence alignment of Rpo41 from selected Ascomycetes (*Bb, Beauveria bassiana; Cm, Cordyceps militaris; Ma, Metarhizium acridium; Mb, Metarhizium brunneum; Fo, Fusarium oxysporum; Fp, Fusarium pseudograminearum; Nh, Nectria haematococca; Vd, Verticillium dahlia; Nc, Neurospora crassa; Pd, Penicillium digitatum; Pc, Penicillium chrysogenum; An, Aspergilus nidulans; Ac, Ajellomyces capsulatum; Sc, Saccharomyces cerevisiae; Ca, Candida albicans; Sp., Schizosaccharomyces pombe*), for determining conserved regions—domains, using ClustalX. **(B)** The amplified whole gene *rpo*41 of *M. brunneum* ARSEEF 3297. **(C)** Crystallographic structure of RNA polymerase is bacteriophage T7 (PDB_ 1QLN). Green represented alpha-helices, while red beta-sheets. The random coil structures are displayed in cyan. The greater part of the protein structure consists of a-helices. **(D)** Prediction of the structure of mt-RNA polymerase (Rpo41p) of *M. brunneum* ARSEF 3297. All structures were constructed using the softwares PSIPRED, HHpred and PyMol. **(E)** A comparative schematic representation of all RNA polymerases and their transcriptional factors as described for T3/T7 phages, archaea, bacteria, mitochondria, and nucleus of eukaryotes (based on information provided by Werner, 2007, 2012, Werner and Grohmann, 2011).

**FIGURE 6:**
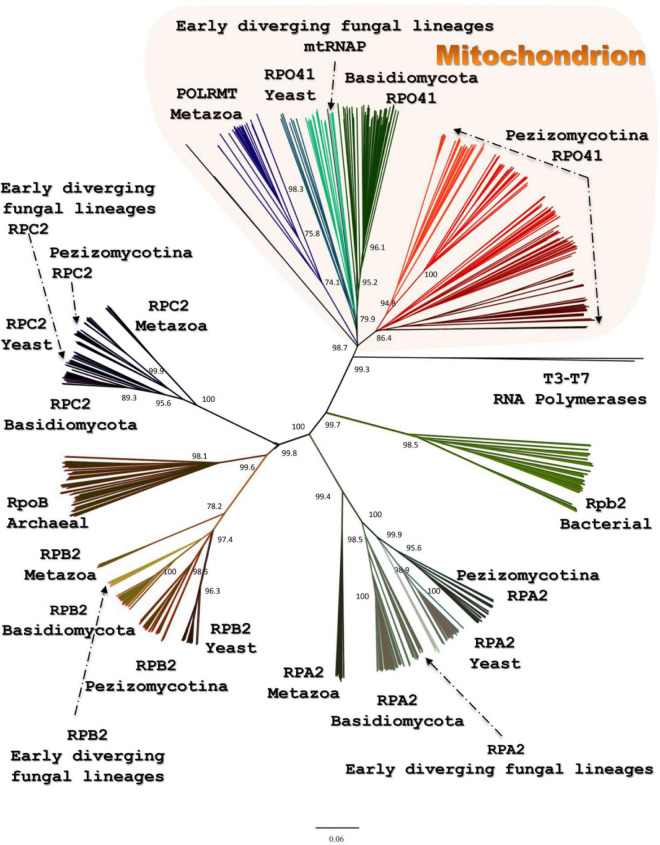
The phylogenetic tree produced for Rpo41 and all homologs RNA polymerases from representatives of all living domains, using NJ and RaxML methods. Numbers at the nodes denote NJ-bootstrap support. RAxML-bootstrap was in all topologies above 90% and it is not shown.

All the above transcriptional elements and features, i.e., promoters, maturation signals, and primary polycistronic transcripts, are of utmost importance for the harmonious function of the mt RNA polymerase and its transcription factor MTF1. Both proteins are encoded by genes located in the nucleus of the species examined. Mt RNA polymerase (Rpo41) was found in contigs PPTI01000001.1 and AZNG01000005.1 of the genomes of *B. bassiana* and *M. brunneum* species (WGS projects: PPTI01 and AZNG01), respectively. Based on these sequences, primers were designed in order to amplify the whole gene in both species (Figure 5). Their sequences were identical to the corresponding gene sequences found in the WGS and thus, it was possible in all cases to identify (a) the mitochondrial target sequence and (b) the conserved functional regions of these proteins (Figure 5A). Especially in the case of Rpo41 of *M. brunneum* ARSEF 3297, the tertiary structure of the protein was built, and all expected conserved regions (Figure 5C) were identified (Figure 5D). The phylogenetic analysis performed in this work including representative sequences of RNA polymerases from all living organisms, showed that the Rpo41 proteins of the Hypocrealean entomopathogenic species cluster with their mitochondrial homologs from Pezizomycotina, and subsequently with other fungal subphyla and then with their counterparts from Metazoa (Figure 6). Their ancestral homolog seems to be the RNA polymerases found at phages and most specifically T7 or T3 phages, which are also related to the bacterial RNA polymerase subunit b (Figure 6). These enzymes are the ancestors of archaeal RpoB and all eukaryotic nuclear RNA polymerases like RPA2, RPB2, and RPC2 (Figure 6).

**FIGURE 7:**
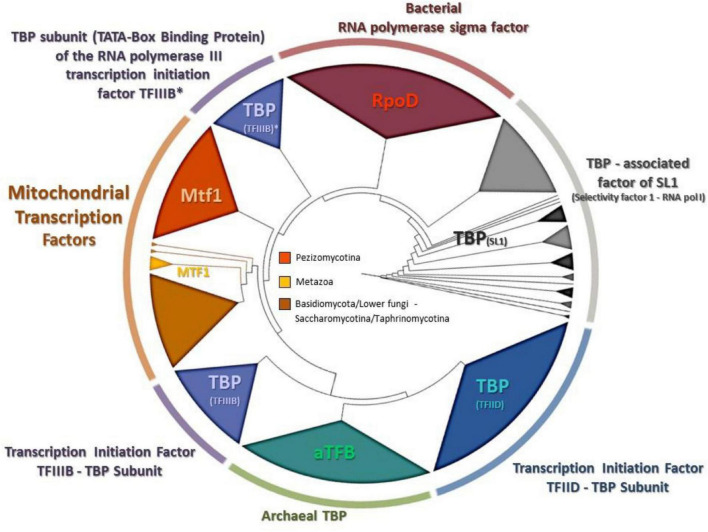
The phylogenetic tree produced for MTF1 and all homologous transcription factors from representatives of all living domains, using NJ and RaxML methods. RAxML-bootstrap support was in all topologies above 95% and it is not shown. Gene clusters of organisms belonging to the same taxonomic division are shown in different colors.

Since the phylogenetic topologies of Rpo41 with the rest RNA polymerases showed a preferred but still relaxed association with their bacterial counterpart (Figure 6), it became evident that the phylogenetic history of the only other needed protein for a functional Rpo41, i.e., Mtf1, had to be studied. It is known that Mtf1 protein assembles with the mitochondrial polymerase in order the latter to be functional and moreover, Mtf1 is similar to the bacterial homolog of sigma factor (Cliften et al., 2000). However, the obtained phylogeny (Figure 7) indicated that MTF1 showed relationship with Transcription Binding Protein (TFIII subunit B) and only basally to the RpoD of bacteria.

**FIGURE 8:**
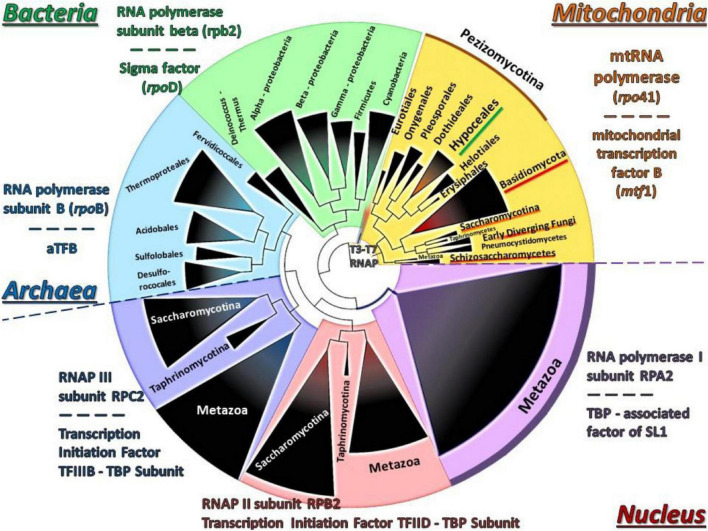
The phylogenetic tree of the concatenated Rpo41-Mtf1 matrix using NJ and RAxML methods. RAxML-bootstrap support was in all topologies above 95% and it is not shown. Gene clusters of organisms belonging to the same taxonomic division are shown in different colors.

Moreover, since the evolution of both Rpo41 and Mtf1 proteins was under investigation, a concatenated matrix of these proteins and their nuclear and prokaryotic counterparts from representative species was created, resulting to a phylogenetic tree (Figure 8). This tree showed that their positioning was basal along with the T7/T3 phage RNA polymerases.

Furthermore, both Rpo41 and Mtf1 of both species examined in this work had all important amino acids which play role to their activity but also conformation conserved, the based on the analysis from ET-viewer (Supplementary Table 4). In addition, the network relationships of each protein examined under the STRING method showed always the greatest hit with its complementary, i.e., Rpo41 vs. Mtf1 and vice versa, irrelevant to the organism from which they were acquired (Supplementary Table 5). Finally, the last method applied, Mirror Tree showed that both trees present almost identical topologies when compared (Supplementary Figure 6). All methods provided strong indications that these two proteins co-evolved.

## Discussion

In this study an effort was made to decipher the mitochondrial transcription of the Hypocrealean entomopathogenic species *B. bassiana* and *M. brunneum*, and correlate any insights acquired with the evolutionary history of the mt transcription and its key proteins. In yeasts, many different studies have identified the mt promoter sequences, their role in the mt transcription and their distribution within the mt genome (Christianson and Rabinowitz, 1983; Biswas et al., 1985; Schinkel et al., 1986; Biswas, 1999; Schäfer et al., 2005; Kim et al., 2012; Deshpande and Patel, 2014). On the other hand, the knowledge concerning mt promoters for species belonging to Pezizomycotina is scarce, with the sole exception of *Neurospora crassa* (Burger et al., 1985; de Vries et al., 1985; Kubelik et al., 1990; Kleidon et al., 2003). Recently, a prediction of putative transcription starting sites for the mt genes of *M. anisopliae* was published (Bantihun and Kebede, 2021). In that study, the analyses were performed *in silico*, whereas in the present work, an emphasis was given on data based both on an *in silico* and experimental evidence, which was further combined with experimental data of the mt gene promoters of yeasts and *N. crassa*, mentioned above. Thus, the common characteristics of mt promoters are (a) their dispersal throughout the genome, (b) their participation in the transcription of polycistronic molecules which are further maturated into single transcripts and (c) their A-T rich content. In this study, both the *in silico* and the experimental data confirmed the above-mentioned characteristics of the mt promoters. In accordance to their phylogenetically closest species, i.e., *N. crassa*, the mt promoter sequence analyses of both entomopathogenic fungi of this work, presented differences to the respective sequence of yeasts. However, a few but distinctive differences of the consensus sequence from all Hypocreales were detected compared to the ones of *N. crassa*, which belongs to the Order Sordariales, especially in the 3′end of the promoter sequence. More specifically there was an underrepresentation of G in the hypocrealean mt putative consensus promoter sequence (Figure 1). It is worth mentioning that *in silico* prediction of the mt promoters is still difficult to be achieved, for the following reasons: (a) fungal mt genomes are AT-rich (Gray et al., 1999; Kouvelis et al., 2004), (b) gene order is variable among species of different Orders, let alone different subphyla (Kouvelis et al., 2004; Pantou et al., 2008), and (c) mt intergenic regions are variable in size and content, even between species or strains of the same genus or species, respectively (Kortsinoglou et al., 2019; Theelen et al., 2021). However, it is shown in this work, that the present abundance of sequenced mt genomes from a large number of species belonging to the same Order, like Hypocreales in this case, provides a solution for an *in silico* approach with good prediction results for determining the sequence of the mt promoter sequences. Moreover, the experimental verification of these predictions with the combined use, primarily, of RT-PCR, secondarily, of Northern Hybridization and thirdly, of 5′ end-RACE PCR, as presented in this study, showed that they may be the most suitable approaches for future similar quests. Still, many difficulties which may burden the results, have been found in this work as well as in previous studies (Burger et al., 1985; de Vries et al., 1985; Bittner-Eddy et al., 1994; Schäfer et al., 2005). In detail, mt polycistronic molecules are under the mechanism of maturation to single transcripts (Bittner-Eddy et al., 1994; Schäfer et al., 2005) and as described earlier, Northern hybridization experiments failed to provide large in size signals, as transcripts are most probably processed before transcription termination (Burger et al., 1985; de Vries et al., 1985). However, Kleidon et al. (2003) have shown that long transcripts may be retained for considerable time periods which allow their detection. RT-PCR and Northern results of this study further verify their conclusion and moreover, may provide indications about the maturation patterns of the polycistronic transcripts (Supplementary Figure 2). It was also shown that mt promoters of Hypocrealean fungal species with their AT-rich composition resemble the prokaryotic −10 element and the eukaryotic (nuclear) TATA box in similar evolutionary studies found in other fungi like *N. crassa* (Kleidon et al., 2003). The position of the mt promoters and the transcriptional start sites as found in this work present a resemblance to the respective elements of bacteriophage genes (Jang and Jaehning, 1991). The results of promoter sequence and the polycistronic transcripts verified the expected similarity of the mt transcription mechanism with the respective bacterial one. Therefore, these results coincide with the endosymbiotic origin of the mitochondria and their genomes (review Martin et al., 2015 and references therein).

A paradox which remains still unanswered is the origin and evolution of the mtRNA polymerase Rpo41. It is well established that the respective nuclear DNA-dependent RNA polymerases are of archaeal origin (see review Kwapisz et al. (2008) and references therein). According to the most widely accepted endosymbiotic theory of an alpha-proteobacterium as the ancestor of the mitochondrion which became a symbiont to an archaeal progenitor (reviews: Gray et al., 1999; Martin et al., 2015 and references therein), mtRNAP seems to be a descendant of a T7 phage progenitor and not of the bacterial counterpart (see review Shutt and Gray (2006) and references therein). Recently, it was shown that the C-terminal domain of Rpo41 presents homology to the catalytic domain of T7 RNAP and partially to the N-terminal domain, but it also contains a 300 amino acid region which has no similarity to the phage enzyme (Yang et al., 2015). Moreover, Rpo41 has few elements in common with the expected bacterial counterpart (Cermakian et al., 1997; Cliften et al., 2000), which are multi-subunit RNA Polymerase (msuRNAPs), closely related to their archaeal and eukaryotic homologs (Cliften et al., 2000). The structural analysis of this enzyme from the Hypocrealean entomopathogenic species of this work (Figure 5 and Supplementary Table 4) and the phylogenetic tree of these enzymes (Figure 6) showed that mt RNA polymerases of all fungi have conserved domains with both phage and bacterial elements. However, the Rpo41 may initiate non-specific transcription, since it has retained all promoter recognition elements found in the T7 RNAp, but in order to produce the correct polycistronic transcripts, the Mtf1 protein is needed (Yang et al., 2015). Mtf1 has all the necessary and critical amino acids found in eubacterial sigma factors, but otherwise limited homology as whole proteins (Cermakian et al., 1997). Therefore, in this study, an effort was made to concatenate both proteins in one matrix (Figure 8), including sequences from representative RNAPs from all domains of life, i.e., phages, archaea, bacteria, eukaryotes (with sequences of both nuclear and mitochondrial origin), in order to define their phylogenetic signal, as it is well established that single genie phylogenies do not always represent the phylogenetic relationships or the evolution of whole organisms or cluster of genes involved in one process (e.g., Pantou et al., 2008; Korovesi et al., 2018). However, single protein phylogenies of this work were in agreement to the previously mentioned studies. Although the tree of the concatenated dataset must be treated cautiously, due to the lack of congruency support, it showed that the fungal holoenzyme of mtRpo41-MTF1 remains basal to the tree and close to the single unit T7/T3 RNA polymerase which does not need any factor for the transcription of its genome (Figure 8). Moreover, based on the review of de Juan et al. (2013), three different methodologies, i.e., the evolutionary trace (SDP method), STRING (method of phylogenetic profiles), and the mirror tree (an inter-protein co-evolution method), were employed and verified that these proteins remained conserved and co-evolved through evolution, despite their differential origin.

A possible mechanism for the acquisition and usage of the Rpo41, the homolog ssuRNAP of T3/T7 phage, instead of the original multi-subunit bacteria-like RNA polymerase, is the inheritance of a bacteriophage T3/T7-like RNA polymerase at the proto-mitochondrial ancestor in a later (secondarily) stage (Burger and Lang, 2003). While this hypothesis cannot be ruled out, in this work phylogenetic analyses, as well as the comparative enzyme structure analyses of these critical to mt transcription proteins, i.e., Rpo41 and MTF1, showed the following: (a) the phylogenetic positioning of the main transcriptional factor MTF1 confirmed the above results as it was shown that MTF1, due to its sequence alignment, remains sister clade to the transcription factors of bacteria which recognize −10 sequences and the eukaryotic factors which need TATA boxes in order to proceed to transcription of the genes (Figure 7), (b) the resemblance of mtRPO41 with the T7/T3 respective enzyme, based on this analyses of an immense number of sequences compared to the original studies was verified (Figures 5, 6 and Supplementary Tables 4, 5), and (c) the presence of both enzymes in all genomes analyzed, a most probable result of vertical inheritance from the early evolution of the eukaryote. Thus, we may support that during the endosymbiotic incident that led to the genesis of the mitochondrion, the progenitor of the mitochondria might have been an ancestral bacterium which was infected with a phage-like progenitor. This hypothesis has been also considered as the most plausible scenario for explaining the role of this phage-like polymerase in mitochondria (Filée and Forterre, 2005), and may be considered as a variation of the “Viral Eukaryogenesis” (VE) hypothesis, proposed by Bell (2001, 2006) and Forterre (2006). However, VE hypothesis proposes that the first eukaryotic cell was a “simultaneous” consortium of three organisms i.e., an archaeon, an a-proteobacterium and a viral ancestor of the nucleus (Bell, 2009). Additionally, in its current version, the unique features of the nucleus, like the uncoupling of transcription from translation, are of viral origin (Bell, 2020). Even when the objections (Koonin and Yutin, 2018; Krupovic et al., 2019; Slijepcevic, 2021) for the acceptance of this hypothesis are considered, our hypothesis of a mitochondrion originating from an a-proteobacterial ancestor infected by a phage-like progenitor cannot be overruled and seems an alternative new variation of the prophage infected alpha-proteobacerium which acted as the mitochondrial progenitor (Filée and Forterre, 2005). Additionally, the results of this work based on the parallel genetic analyses of the genomes’ transcriptional elements and the phylogenetic analyses of transcription’s key enzymes (as presented above) are in accordance to results of previous studies (Masters et al., 1987; Tracy and Stern, 1995; Cliften et al., 2000), thus supporting further the hypothesis proposed by Filée and Forterre (2005). This work further improves this latter hypothesis by pinpointing the co-operation of phage (RNA polymerase) and a bacterial (sigma-like Mtf1) originated components for the correct transcription of mt genes. The alternative possible hypothesis that the acquisition of an ancestral polymerase related to phages is a result of Horizontal Gene Transfer is weak based on the data of this work, since there are indications mentioned above which show that the mt polymerase and transcription factor were included in the mt gene transcription from the beginning and coevolved from the early start of the mitochondrion’s formation.

## Conclusion

In conclusion, the mt transcription of the fungal entomopathogenic species *B. bassiana* and *M. brunneum* was analyzed in this study, concerning the promoters involved, the polycistronic transcripts which are transcribed and their possible maturation, the structure and phylogeny of the key enzymes of this transcription, i.e., Rpo41 and MTF1. It was shown that there is a certain diversity of the promoter sites and of the content of the polycistronic transcripts among the different species, especially between yeasts and Pezizomycotina. However, a consensus is obvious to the key enzymes and the mechanism of transcription. If the phylogenetic analyses are expanded to all RNA polymerases and their transcriptional factors from representatives from all domains of life, then this comparative analysis provides insights to the evolution of the mitochondrion and the mechanism of transcription of its genome. In detail, it suggests that the mechanism of fungal mt transcription is similar to the bacterial respective process with the inclusion of a single transcription factor. The RNA polymerase is of phage origin and, as suggested previously by other studies, might have been acquired in a very early stage of the mitochondrion’s formation. That is, the bacterial endosymbiont was infected with a phage and for reasons which need further examination, this polymerase of the phage ancestor remained in the mitochondrion progenitor as the functional one. This polymerase, however, coevolved further with the existing primitive sigma factor which evolved to the MTF1 factor.

## Data Availability Statement

The datasets presented in this study can be found in online repositories. The names of the repository/repositories and accession number(s) can be found in the article/Supplementary Material.

## Author Contributions

SV and VK conceived, designed the study, and analyzed the data. SV conducted the experiments and wrote the manuscript. VK revised the manuscript. Both authors read and approved the final manuscript.

## Conflict of Interest

The authors declare that the research was conducted in the absence of any commercial or financial relationships that could be construed as a potential conflict of interest.

## Publisher’s Note

All claims expressed in this article are solely those of the authors and do not necessarily represent those of their affiliated organizations, or those of the publisher, the editors and the reviewers. Any product that may be evaluated in this article, or claim that may be made by its manufacturer, is not guaranteed or endorsed by the publisher.

## Footnotes

1
https://pymol.org

2
http://tree.bio.ed.ac.uk/software/figtree/

3
https://string-db.org/cgi/input?sessionId=buxdn3JsL4Jy&input_page_show_search=on

4
http://evolution.lichtargelab.org/ETviewer

5
http://csbg.cnb.csic.es/mtserver/#

6
http://phylo.io/index.html

## Supplementary Material

Supplementary Figure 1 The map of the mitochondrial genome of *Metarhizium brunneum* ARSEF 3297. Arrows indicate the direction of gene transcription. The inner circles show the GC content. All genes identified are indicated in italics.

Supplementary Figure 2 Northern blots of mitochondrial transcriptome and gene synteny of mt genome for *B. bassiana*. RNA gel−blot hybridizations with comprehensive sets of labeled oligonucleotides covering the sense DNA strand in *B. bassiana* ATHUM 4946: (I) *rnl*, (II) *nad*3, (III) *cob*, (IV) *cox*1, (V) *nad*4, (VI) *cox*3. Inferred processing pathway for transcripts of the polycistronic transcription units in mitochondria from *B. bassiana*. The above pathways result from an analysis of the transcripts that behave as cohorts (six polycistronic transcription units) and are detected by related hybridization probes, in RNA samples from spores at different stages of germination. The triangle symbol denotes a nuclease processing step that occurs for the gene-specific post-transcriptional mechanism in the expression of protein-coding genes in Hypocrealean mitochondria. Transcripts, exons and introns are not drawn to scale. The direction of transcription is from left to right.

Supplementary Figure 3 The phylogenetic tree produced for Rpo41 and all homologs RNA polymerases from representatives of all living domains, using NJ and RaxML methods. Numbers at the nodes denote NJ-bootstrap support. RAxML-bootstrap was in all topologies above 90% and it is not shown. OTUs are presented as acronyms whose full details are provided in Supplementary Table 3.

Supplementary Figure 4 The phylogenetic tree produced for MTF1 and all homologous transcription factors from representatives of all living domains, using NJ and RaxML methods. RAxML-bootstrap support was in all topologies above 95% and it is not shown. Gene clusters of organisms belonging to the same taxonomic division are shown in different colors. OTUs are presented as acronyms whose full details are provided in Supplementary Table 3.

Supplementary Figure 5 The phylogenetic tree of the concatenated Rpo41-Mtf1 matrix using NJ and RAxML methods. RAxML-bootstrap support was in all topologies above 95% and it is not shown. Gene clusters of organisms belonging to the same taxonomic division are shown in different colors. OTUs are presented as acronyms whose full details are provided in Supplementary Table 3.

Supplementary Figure 6 The comparison of the two single phylogenetic trees of Rpo41 and Mtf1 as produced by MirrorTree and showed by Phylo.io. The species included are shown in numbers (according to the enumeration of species in PBD databank) as follows: *Ashbya gossypii* 284811, *Candida glabrata* 284593, *Kluyveromyces lactis* 284590, *Komagataella phaffii* 644223, *Lachancea thermotolerans* 559295, *Meyerozyma guillermondii* 294746, *S. cerevisiae* 285006, 307796, 559292, 574961, 643680, and *Zygosaccharomyces rouxii* 559307.

Supplementary Table 1 The annotated complete mt-genome of *M. brunneum* ARSEF 3297.

Supplementary Table 2 Annotation attributes of the complete mt genomes of *M. brunneum* strains ARSEF 3297 and ARSEF 4556. Cells highlighted in yellow indicate intronic ORFs. Genes encoding rRNAs, and subunits of ATP synthase, Apocytochrome b, Cytochrome oxidase c and NADH dehydrogenase are shown as highlighted cells in blue, orange, light gray, purple and light green, respectively.

Supplementary Table 3 The representative species from all domains of life for which the RNA polymerases and their Transcription factors were used in the phylogenetic analyses. Their taxonomic status of these species, the GenBank Accession Numbers of their sequences are provided.

Supplementary Table 4 Conserved important residues of Rpo41 and Mtf1 proteins as presented by ET1052viewer and the analyses of De Wijngaert et al., 2021.

Supplementary Table 5 Predicted Complex Interaction of Rpo41 and MTF1 as found by STRING method.
