# Supplementary material for: Mitochondrial Transcription of Entomopathogenic Fungi Reveals Evolutionary Aspects of Mitogenomes
Source: Front Microbiol. 2022 Mar 21;13:821638. doi: 10.3389/fmicb.2022.821638 (PMC8979003; doi:10.3389/fmicb.2022.821638)
Supplement: Supplementary Figure 1 — The map of the mitochondrial genome of Metarhizium brunneum ARSEF 3297. Arrows indicate the direction of gene transcription. The inner circles show the GC content. All genes identified are indicated in italics. [file Data_Sheet_1.zip › Figure S4.pptx]

## Slide 1
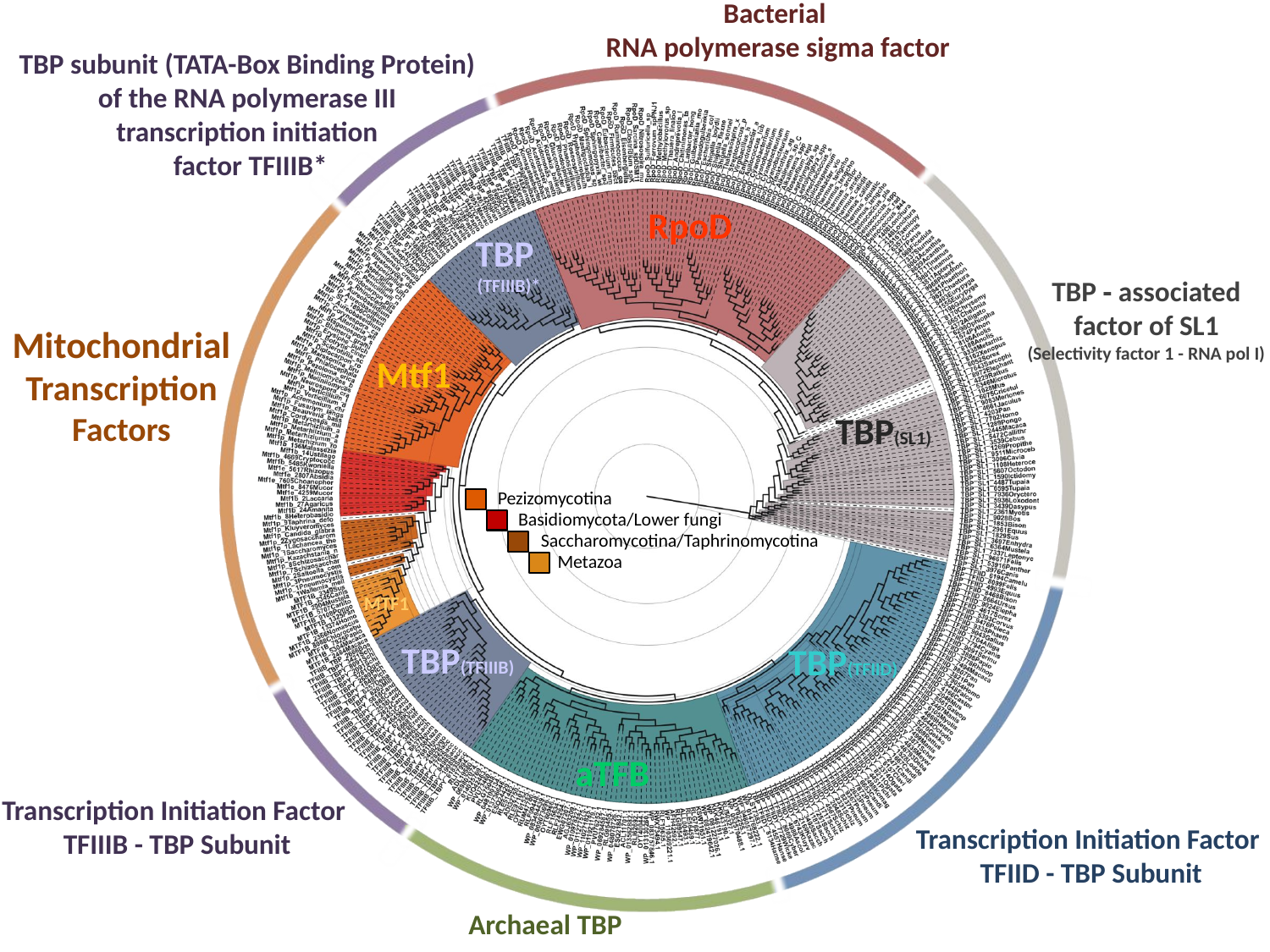

Bacterial
RNA polymerase sigma factor
TBP subunit (TATA-Box Binding Protein)
of the RNA polymerase III
transcription initiation
factor TFIIIB*
RpoD
TBP
 (TFIIIB)*
TBP ‐ associated
factor of SL1
(Selectivity factor 1 - RNA pol I)
Mitochondrial
Transcription
Factors
Mtf1
TBP(SL1)
Pezizomycotina
Basidiomycota/Lower fungi
Saccharomycotina/Taphrinomycotina
Metazoa
MTF1
TBP(TFIIIB)
TBP(TFIID)
aTFB
Transcription Initiation Factor
TFIIIB - TBP Subunit
Transcription Initiation Factor
TFIID - TBP Subunit
Archaeal TBP
