# Supplementary material for: Mitochondrial Transcription of Entomopathogenic Fungi Reveals Evolutionary Aspects of Mitogenomes
Source: Front Microbiol. 2022 Mar 21;13:821638. doi: 10.3389/fmicb.2022.821638 (PMC8979003; doi:10.3389/fmicb.2022.821638)
Supplement: Supplementary Figure 1 — The map of the mitochondrial genome of Metarhizium brunneum ARSEF 3297. Arrows indicate the direction of gene transcription. The inner circles show the GC content. All genes identified are indicated in italics. [file Data_Sheet_1.zip › Figure S5.pptx]

## Slide 1
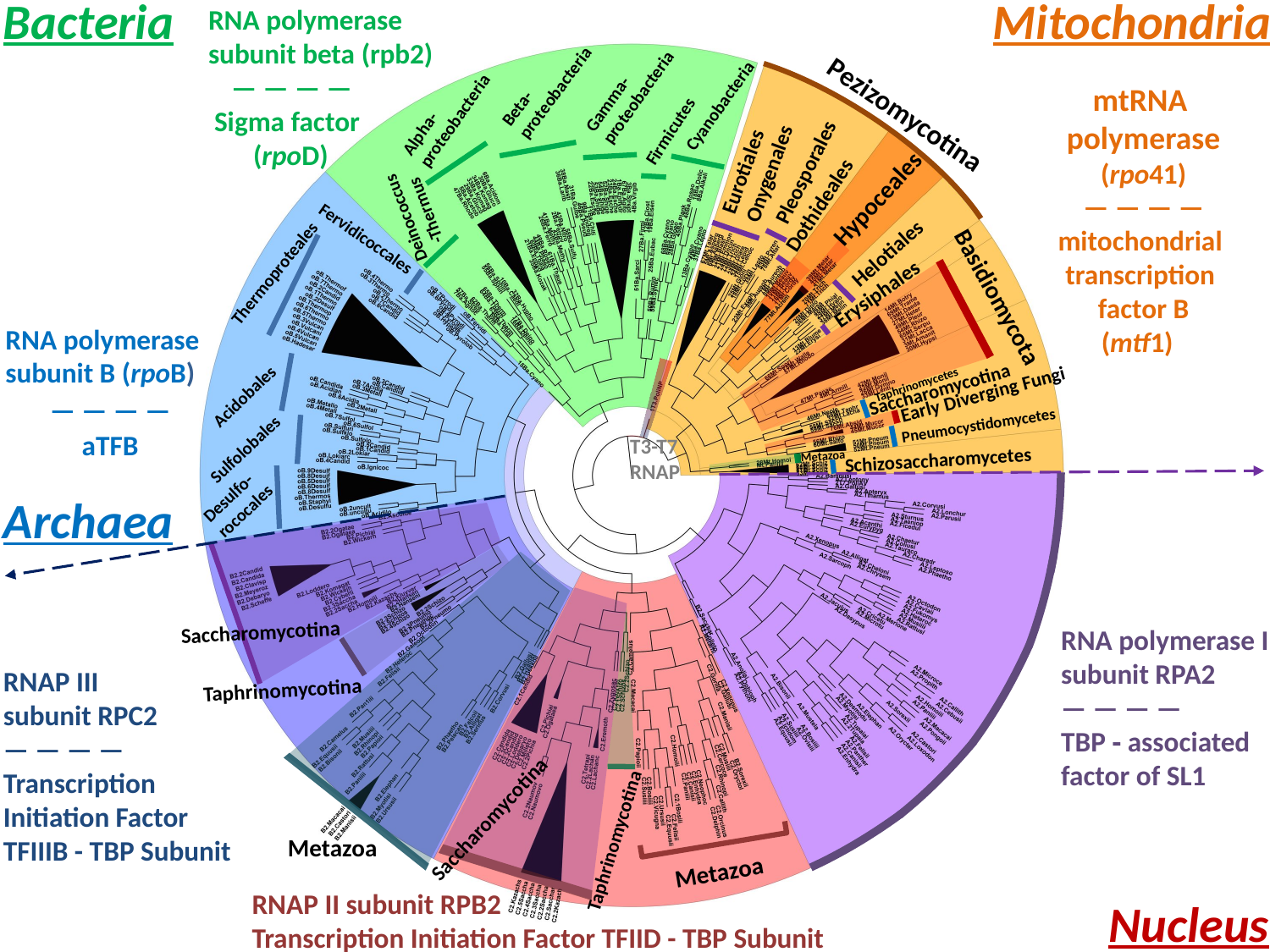

Bacteria
Mitochondria
RNA polymerase
subunit beta (rpb2)
mtRNA
polymerase
(rpo41)
— — — —
mitochondrial
transcription
factor B
(mtf1)
Beta-
proteobacteria
Gamma-
proteobacteria
— — — —
Alpha-
proteobacteria
Cyanobacteria
Pezizomycotina
Sigma factor
(rpoD)
Firmicutes
Eurotiales
Onygenales
Pleosporales
Hypoceales
Dothideales
Deinococcus
-Thermus
Fervidicoccales
Helotiales
Thermoproteales
Erysiphales
Basidiomycota
RNA polymerase
subunit B (rpoB)
Taphrinomycetes
Saccharomycotina
Early Diverging Fungi
Acidobales
— — — —
aTFB
Pneumocystidomycetes
T3-T7
RNAP
Sulfolobales
Schizosaccharomycetes
Metazoa
Desulfo-
rococales
Archaea
Saccharomycotina
RNA polymerase I
subunit RPA2
— — — —
TBP ‐ associated
factor of SL1
RNAP III
subunit RPC2
— — — —
Transcription
Initiation Factor
TFIIIB - TBP Subunit
Taphrinomycotina
Saccharomycotina
Taphrinomycotina
Metazoa
Metazoa
RNAP II subunit RPB2
Transcription Initiation Factor TFIID - TBP Subunit
Nucleus
