# Supplementary material for: Mitochondrial Transcription of Entomopathogenic Fungi Reveals Evolutionary Aspects of Mitogenomes
Source: Front Microbiol. 2022 Mar 21;13:821638. doi: 10.3389/fmicb.2022.821638 (PMC8979003; doi:10.3389/fmicb.2022.821638)
Supplement: Supplementary Figure 1 — The map of the mitochondrial genome of Metarhizium brunneum ARSEF 3297. Arrows indicate the direction of gene transcription. The inner circles show the GC content. All genes identified are indicated in italics. [file Data_Sheet_1.zip › Figure S6.docx]

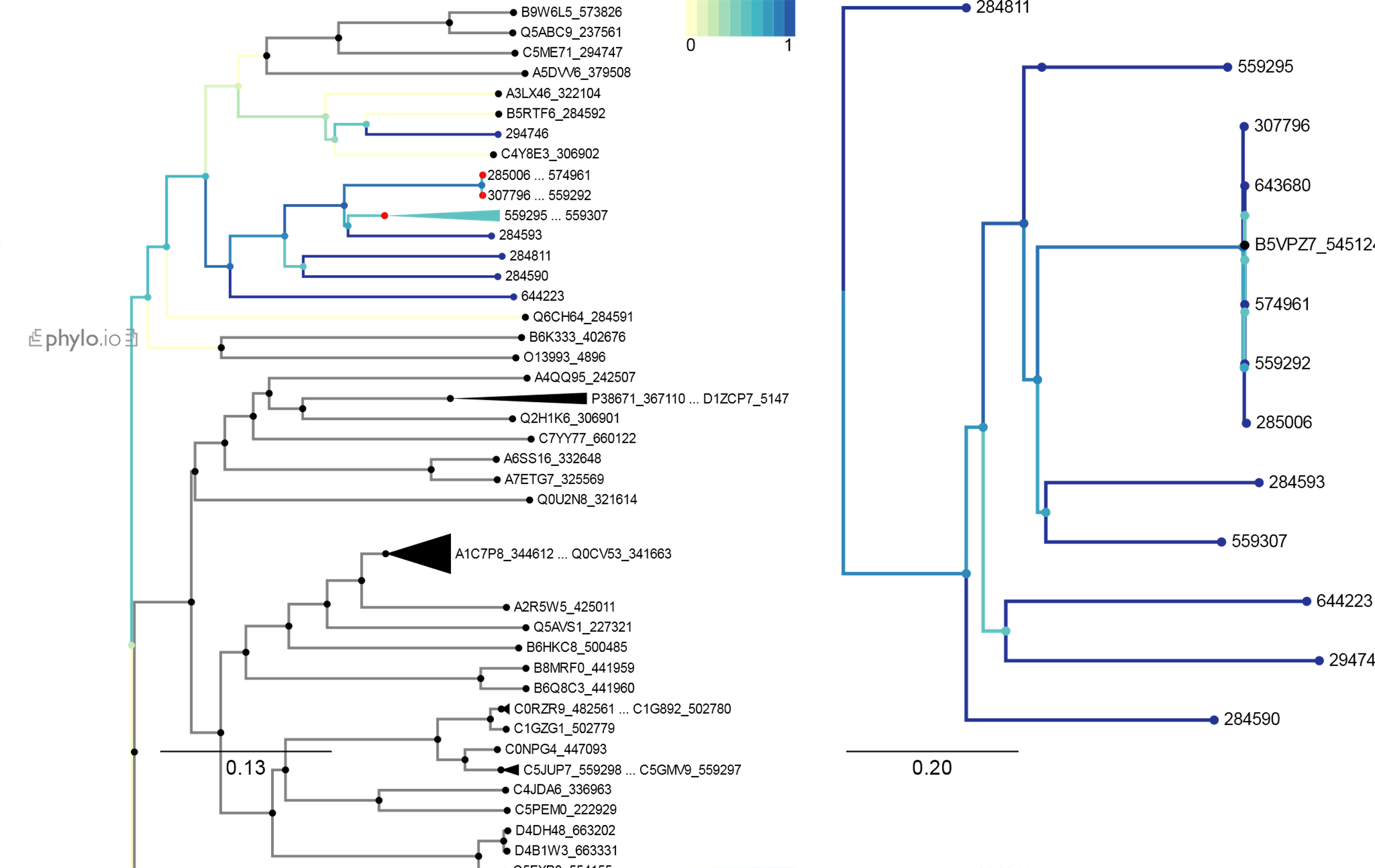

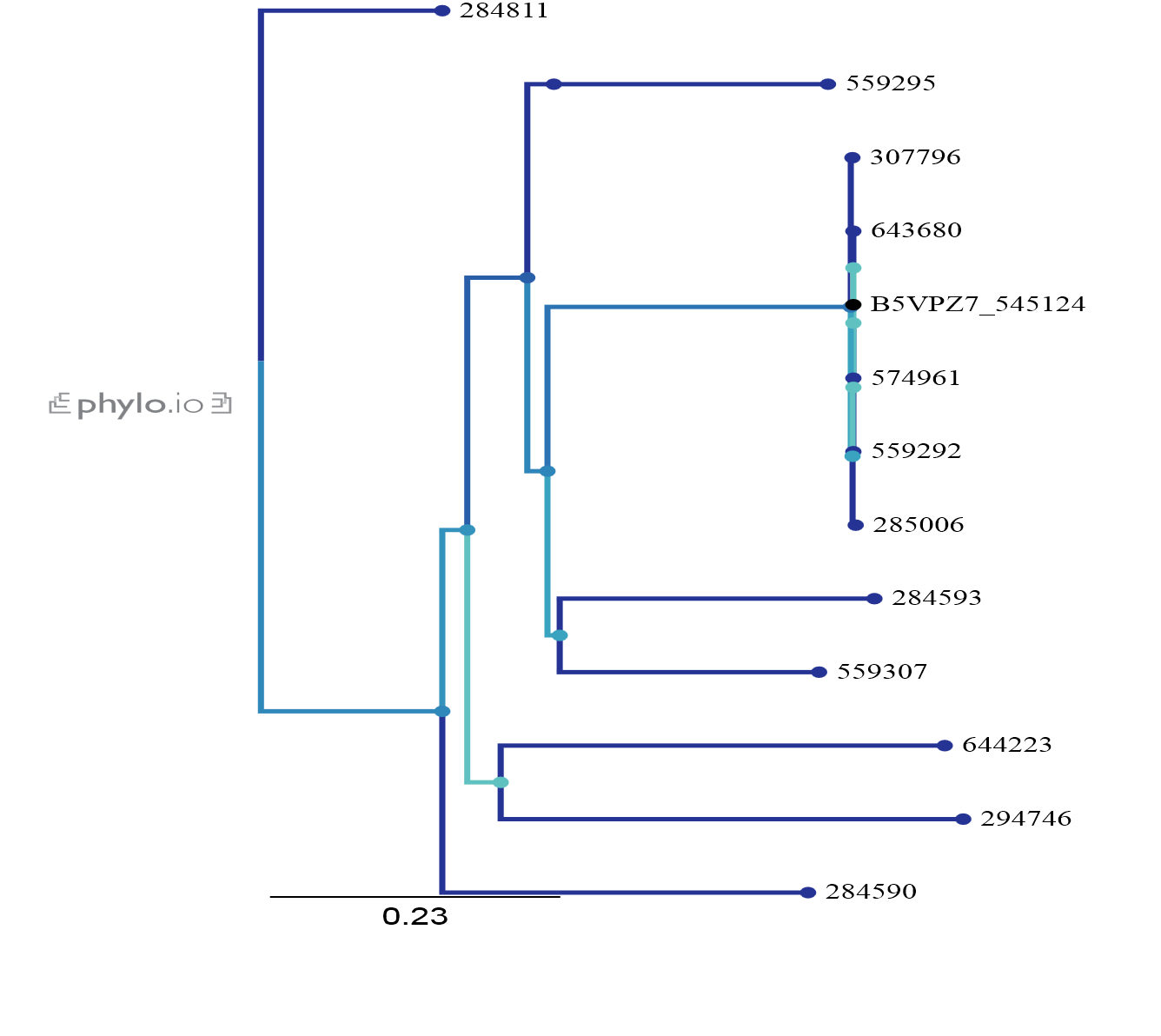


**Supplementary Figure S6.** The comparison of the two single phylogenetic trees of Rpo41 and Mtf1 as produced by MirrorTree and showed by Phylo.io. The species included are shown in numbers (according to the enumeration of species in PBD databank) as follows: *Ashbya gossypii* 284811, *Candida glabrata* 284593, *Kluyveromyces lactis* 284590, *Komagataella phaffii* 644223, *Lachancea thermotolerans* 559295, *Meyerozyma guillermondii* 294746, *S. cerevisiae* 285006, 307796, 559292, 574961, 643680 and *Zygosaccharomyces rouxii* 559307.
