# Supplementary figures and images for: Mitochondrial Transcription of Entomopathogenic Fungi Reveals Evolutionary Aspects of Mitogenomes
Source: Front Microbiol. 2022 Mar 21;13:821638. doi: 10.3389/fmicb.2022.821638 (PMC8979003; doi:10.3389/fmicb.2022.821638)

**Figure S1**.


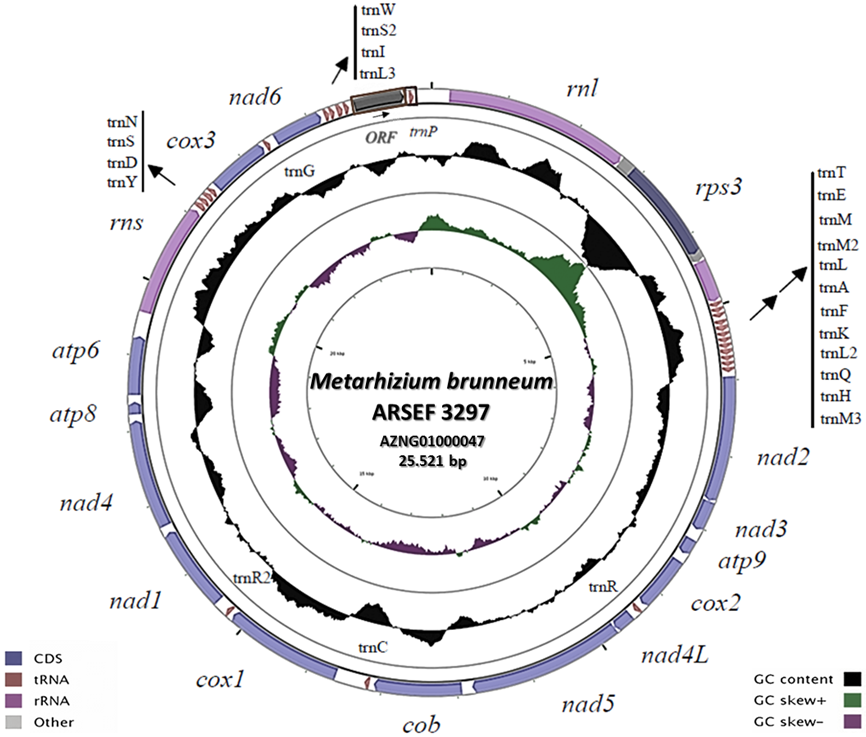

Supplement: Supplementary Figure 1 — The map of the mitochondrial genome of Metarhizium brunneum ARSEF 3297. Arrows indicate the direction of gene transcription. The inner circles show the GC content. All genes identified are indicated in italics. [file Data_Sheet_1.zip › Figure S1.docx]

**Figure S2**.


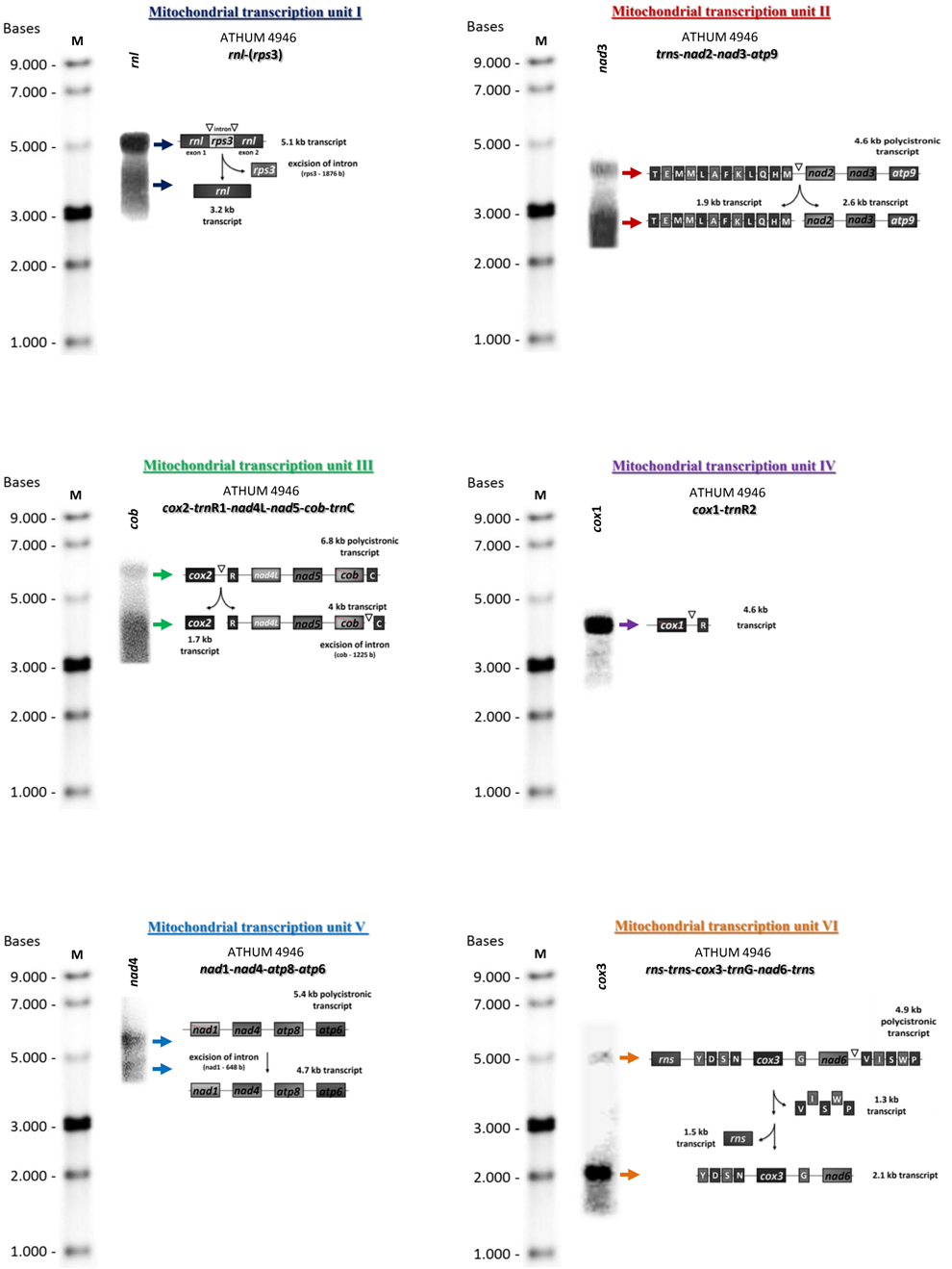

Supplement: Supplementary Figure 1 — The map of the mitochondrial genome of Metarhizium brunneum ARSEF 3297. Arrows indicate the direction of gene transcription. The inner circles show the GC content. All genes identified are indicated in italics. [file Data_Sheet_1.zip › Figure S2.docx]
